# Identification of potential novel N6-methyladenosine effector-related lncRNA biomarkers for serous ovarian carcinoma: a machine learning-based exploration in the framework of 3P medicine

**DOI:** 10.3389/fphar.2024.1351929

**Published:** 2024-06-04

**Authors:** Lele Ye, Xinya Tong, Kan Pan, Xinyu Shi, Binbing Xu, Xuyang Yao, Linpei Zhuo, Su Fang, Sangsang Tang, Zhuofeng Jiang, Xiangyang Xue, Weiguo Lu, Gangqiang Guo

**Affiliations:** ^1^ Wenzhou Collaborative Innovation Center of Gastrointestinal Cancer in Basic Research and Precision Medicine, Wenzhou Key Laboratory of Cancer-Related Pathogens and Immunity, Department of Microbiology and Immunology, Institute of Molecular Virology and Immunology, Institute of Tropical Medicine, School of Basic Medical Sciences, Wenzhou Medical University, Wenzhou, Zhejiang, China; ^2^ Women’s Reproductive Health Laboratory of Zhejiang Province, Women’s Hospital, Zhejiang University School of Medicine, Hangzhou, Zhejiang, China; ^3^ First Clinical College, Wenzhou Medical University, Wenzhou, Zhejiang, China; ^4^ Institute of Immunology, Zhejiang University School of Medicine, Hangzhou, Zhejiang, China; ^5^ Haiyuan College, Kunming Medical University, Kunming, Yunnan, China; ^6^ Department of Biochemistry, School of Medicine, Southern University of Science and Technology, Shenzhen, Guangdong, China; ^7^ Department of Gynecologic Oncology, Women’s Hospital, Zhejiang University School of Medicine, Hangzhou, Zhejiang, China; ^8^ Center of Uterine Cancer Diagnosis and Therapy of Zhejiang Province, Hangzhou, Zhejiang, China

**Keywords:** m^6^A modification, immunotherapy, biomarker, RP11-508M8.1, predictive, preventive, personalized medicine (PPPM/3PM)

## Abstract

**Background:**

Serous ovarian carcinoma (SOC) is considered the most lethal gynecological malignancy. The current lack of reliable prognostic biomarkers for SOC reduces the efficacy of predictive, preventive, and personalized medicine (PPPM/3PM) in patients with SOC, leading to unsatisfactory therapeutic outcomes. N6-methyladenosine (m^6^A) modification-associated long noncoding RNAs (lncRNAs) are effective predictors of SOC. In this study, an effective risk prediction model for SOC was constructed based on m^6^A modification-associated lncRNAs.

**Methods:**

Transcriptomic data and clinical information of patients with SOC were downloaded from The Cancer Genome Atlas. Candidate lncRNAs were identified using univariate and multivariate and least absolute shrinkage and selection operator-penalized Cox regression analyses. The molecular mechanisms of m^6^A effector-related lncRNAs were explored via Gene Ontology, pathway analysis, gene set enrichment analysis, and gene set variation analysis (GSVA). The extent of immune cell infiltration was assessed using various algorithms, including CIBERSORT, Microenvironment Cell Populations counter, xCell, European Prospective Investigation into Cancer and Nutrition, and GSVA. The calcPhenotype algorithm was used to predict responses to the drugs commonly used in ovarian carcinoma therapy. *In vitro* experiments, such as migration and invasion Transwell assays, wound healing assays, and dot blot assays, were conducted to elucidate the functional roles of candidate lncRNAs.

**Results:**

Six m^6^A effector-related lncRNAs that were markedly associated with prognosis were used to establish an m^6^A effector-related lncRNA risk model (m^6^A-LRM) for SOC. Immune microenvironment analysis suggested that the high-risk group exhibited a proinflammatory state and displayed increased sensitivity to immunotherapy. A nomogram was constructed with the m^6^A effector-related lncRNAs to assess the prognostic value of the model. Sixteen drugs potentially targeting m^6^A effector-related lncRNAs were identified. Furthermore, we developed an online web application for clinicians and researchers (https://leley.shinyapps.io/OC_m6A_lnc/). Overexpression of the lncRNA RP11-508M8.1 promoted SOC cell migration and invasion. *METTL3* is an upstream regulator of RP11-508M8.1. The preliminary regulatory axis METTL3/m^6^A/RP11-508M8.1/hsa-miR-1270/ARSD underlying SOC was identified via a combination of *in vitro* and bioinformatic analyses.

**Conclusion:**

In this study, we propose an innovative prognostic risk model and provide novel insights into the mechanism underlying the role of m^6^A-related lncRNAs in SOC. Incorporating the m^6^A-LRM into PPPM may help identify high-risk patients and personalize treatment as early as possible.

## 1 Introduction

Ovarian carcinoma (OC) is the most lethal gynecological cancer, with serous ovarian carcinoma (SOC) accounting for most of the reported OC cases (Kotsopoulos et al., 2014; Bowtell et al., 2015). Most patients with SOC are diagnosed at an advanced stage due to the concealed anatomical location of the ovaries and the absence of obvious or specific early clinical symptoms. High recurrence rates and drug resistance lead to poor prognoses for patients with SOC (Siegel et al., 2019). Given the complexity, heterogeneity, and refractory nature of SOC, using predictive, preventive, personalized medicine (PPPM/3PM) may help predict patient prognosis, identify tumor characteristics, and optimize treatment plans. PPPM has become a research hotspot in precision cancer medicine, especially, multi-omics and network-based search for prognostic markers that may facilitate accurate diagnosis and treatment (Cheng and Zhan, 2017). However, the outcomes of PPPM for SOC remain unsatisfactory.

Recent advances in immunotherapy, as exemplified by the use of immune checkpoint inhibitors (ICIs), has resulted in its incorporation into the treatment regimens for a range of advanced cancers (Murciano-Goroff et al., 2020; Zhang and Zhang, 2020). The degree of immune cell infiltration into the tumor microenvironment (TME) is strongly associated with the efficacy of cancer immunotherapy (Binnewies et al., 2018; Duan et al., 2020). Currently, the spatial distribution of tumor-infiltrating immune cells is used to classify tumors as “hot tumors,” which are sensitive to immunotherapy (such as those presenting an immune-inflamed phenotype), and “cold tumors,” which are less sensitive to immunotherapy (such as those presenting immune-excluded and immune-desert phenotypes) (Duan et al., 2020; Liu and Sun, 2021). The landscape of SOC is complex and demonstrates potential immunogenicity (Yang et al., 2020; Morand et al., 2021). Nevertheless, the rate of response to immunotherapy in SOC remains suboptimal, necessitating the identification of ideal biomarkers that would facilitate precise selection of immunotherapy regimens for patients.

Long noncoding RNAs (lncRNAs) are a class of RNA molecules that are longer than 200 nucleotides and have limited or no protein-coding capacity (Liu et al., 2020; Statello et al., 2021). LncRNAs regulate the proliferation, apoptosis, metastasis, and drug resistance of tumor cells (Luo et al., 2017; Peng et al., 2017; Muller et al., 2019; Bhat et al., 2020; Wei et al., 2020), and their abnormal expression is closely associated with the severity of malignancy in various cancers, including SOC. Moreover, research has shown that ncRNAs could have a potential dynamic role in future cancer therapeutics, supporting personalized treatment decisions and modern precision medicine (Soureas et al., 2023). N6-methyladenosine (m^6^A), a dynamic and reversible post-transcriptional modification commonly found on mRNAs and lncRNAs (Chen et al., 2020), is a promising clinically relevant biomarker and therapeutic target (Huang et al., 2016; Zhao and Cui, 2019). It is regulated by m^6^A effectors, such as methyltransferases (i.e., writers), demethyltransferases (i.e., erasers), and m^6^A-binding proteins (i.e., readers) (Fu et al., 2014). Numerous studies have demonstrated that m^6^A and its effectors play an essential role in cellular metabolism (Liu et al., 2019), immunomodulation (Shulman and Stern-Ginossar, 2020), malignant progression of tumors (Hou et al., 2021), and drug resistance (Mehrdad et al., 2023). In addition, several studies have been devoted to the development of small-molecule inhibitors for m^6^A modification to improve the efficacy of chemotherapy, radiotherapy, and immunotherapy (Gu et al., 2020; Deng et al., 2023).

Several studies have reported interactions between m^6^A modifications and lncRNAs (Ma et al., 2019; Yi et al., 2020). m^6^A modifications affect the functions of lncRNAs through an m^6^A-switch, thereby inhibiting transcription, mediating competing endogenous RNA (ceRNA) effects, and regulating lncRNA stability or degradation (Jin and Fan, 2023; Mehrdad et al., 2023); for example, METTL14-mediated m^6^A methylation and TINCR lncRNA regulation in pyroptosis and diabetic cardiomyopathy (Meng et al., 2022). The combination of the m^6^A reader YTHDC1 and lncRNA *XIST* promotes lncRNA *XIST*-mediated gene repression (Patil et al., 2016). Yang et al. found that the m^6^A-modified *linc1281* functions as a ceRNA to sequester let-7 miRNAs, thereby exerting regulatory effects on the differentiation of mouse embryonic stem cells (Yang et al., 2018). The m^6^A eraser ALKBH5 promotes the invasion and metastasis of gastric cancer (GC) by removing the m^6^A modification on the lncRNA NEAT1 (Zhang et al., 2019). In addition, lncRNAs may also regulate the functions of cancer-associated m^6^A effectors (Yi et al., 2020). For example, the interplay between the lncRNA LINC00470 and METTL3 contributes to the advancement of GC by enhancing their interaction with the PTEN mRNA and diminishing its stability (Yan et al., 2020). Wang X. et al. reported that the lncRNA GAS5-AS1 enhances the stability of the tumor suppressor GAS5 by interacting with ALKBH5, which removes m^6^A modification on GAS5, thereby inhibiting the proliferation, migration, and invasion of cervical cancer cells (Wang X. et al., 2019). Additionally, the lncRNA LIN28B-AS1 enhances the stability of *LIN28B* mRNA by interacting with the m^6^A reader IGF2BP1, thereby promoting the proliferation and metastasis of lung adenocarcinoma (Wang C. et al., 2019).

Given the complexity of the mechanisms underlying the interaction between m^6^A modifications and lncRNAs, an increasing number of studies have investigated their potential applications in the diagnosis, prognosis, and treatment of tumors and determining the sensitivity of cancer cells to chemotherapeutic agents (Jin and Fan, 2023). A previous study accurately predicted the 5-year survival of patients with GC by stratifying their overall survival (OS) using a risk prediction model based on 11 m^6^A-associated lncRNAs (Wang H et al., 2021). Similarly, a risk prediction model constructed with m^6^A-associated lncRNAs has been used to effectively assess the prognosis of patients with lung adenocarcinoma and predict their response to immunotherapy (Xu et al., 2021). Furthermore, m^6^A effector-related lncRNAs have also been used to establish prediction models for colon adenocarcinoma (Zhang et al., 2021), clear cell renal cell carcinoma (Qiu et al., 2021), breast cancer (Zhang et al., 2020), and pancreatic ductal adenocarcinoma (Hu and Chen, 2021). Thus, m^6^A effector-related lncRNAs may serve as prognostic biomarkers of various cancers and could potentially guide effective and precise individualized treatment. However, the association between m^6^A effector-related lncRNAs and the diagnosis and prognosis of patients with SOC remains unclear. Further studies on the interactions between m^6^A modification and lncRNAs as well as their biological roles in SOC may help reveal the potential of m^6^A effector-related lncRNAs in PPPM.

In this study, we identified six m^6^A effector-related lncRNAs via Pearson’s correlation, univariate and multivariate Cox regression, and least absolute shrinkage and selection operator (LASSO)-penalized Cox regression analyses using transcriptomic and clinical data of patients with SOC obtained from TCGA database. These six lncRNAs were then used to establish an effective risk prediction model for SOC and develop a web link for clinicians and researchers. Subsequently, we used this newly developed risk model to explore immune-related factors, the TME, and the immunotherapeutic response in SOC. Several drugs capable of potentially targeting m^6^A effector-related lncRNAs were identified. In addition, one risky lncRNA was selected, and its role and correlation with the m^6^A effectors in SOC was explored. Our findings could potentially enhance PPPM implementation, enable target prevention, facilitate prognostic assessment, and provide potential biomarkers that may supplement clinical diagnosis as well as treatment in patients with SOC.

## 2 Materials and methods

### 2.1 Gene expression profiles and clinical information of patients with SOC

Transcriptomic and mutational data of patients with SOC were downloaded from TCGA using the “TCGAbiolinks” package in R, in September 2021. Information regarding the neoantigen load and mutation burden of patients with SOC was downloaded from The Cancer Immunome Atlas database (TCIA, https://tcia.at/). Genes were annotated using the GENCODE database (https://www.gencodegenes.org). Corresponding clinical information was downloaded from the cBioPortal database (https://www.cbioportal.org). Samples with missing OS values were excluded. As previously reported (Xu et al., 2021), data were randomly divided into training and testing sets in a ratio of 6:4 (Supplementary Table S1). The total data were used as the validation set. The expression of genes was normalized using fragments per kilobase of exon model per million mapped fragments.

The m^6^A effectors included 12 m^6^A writers (CBLL1, METTL14, METTL16, METTL3, METTL5, VIRMA, RBM15, RBM15B, TRMT112, WTAP, ZC3H13, and ZCCHC4), 19 m^6^A readers (ELAVL1, EIF3A, FMR1, G3BP1, G3BP2, HNRNPA2B1, HNRNPC, IGF2BP1, IGF2BP2, IGF2BP3, LRPPRC, RBMX, PRRC2A, SND1, YTHDC1, YTHDC2, YTHDF1, YTHDF2, and YTHDF3), and 2 m^6^A erasers (ALKBH5 and FTO), as described in previous studies (Zhang Z. et al., 2021; Wang X et al., 2021; Xu et al., 2021). The gene expression profiles (GEPs) of coding genes (including m^6^A effectors) and lncRNAs needed for subsequent analyses were obtained from TCGA. Pearson’s correlation analysis was performed to determine the association between m^6^A effectors and lncRNAs via “Hmisc” (R package) and visualized using “ggsankey” (R package).

### 2.2 Establishment and validation of a risk score model

The training set was used to construct the m^6^A effector-related lncRNA risk model (m^6^A-LRM). LncRNAs were screened via univariate Cox regression, LASSO Cox regression (using the penalty parameter estimated by 10-fold cross-validation), and multivariate Cox regression analyses using the “survival” and “glmnet” packages in R. Receiver operating characteristic (ROC) curves were analyzed and visualized using the “ROCR” package in R. The prognostic risk score was calculated as follows:

$$\begin{array}{ll} m^{6}A-\text{LRM risk score}= & \text{coefficient}\left(\text{lncRNA}1\right)\times \text{expression}\left(\text{lncRNA}1\right)\\ & +\text{coefficient}\left(\text{lncRNA}2\right)\times \text{expression}\left(\text{lncRNA}2\right)\\ & +\ldots +\text{coefficient}\left(\text{lncRNAn}\right)\\ & \times \text{expression}\left(\text{lncRNAn}\right) \end{array}$$

Patients in the training, testing, and validation sets were divided into low- and high-risk groups based on the cut-off risk score using the “surv_cutpoint” function of the “survminer” package in R. Both the testing and validation sets were used to validate m^6^A-LRM, and the results were visualized using the “survminer” package in R. Univariate and multivariate Cox regression analyses were conducted to evaluate the independent effect of m^6^A-LRM using the “survival” and “survminer” packages in R. Principal components analysis was performed for effective dimensionality reduction, model identification, and grouping using the “prcomp” function and visualized using the “scatterplot3d” package in R. Mutation information was summed, compared, and visualized using the “maftools” package in R. Nomograms were constructed using the “rms” package in R. The results of decision curve analysis and calibration plots were visualized using the “rms” package in R. The time-dependent area under the ROC curve (AUC) was analyzed and visualized using the “timeROC” and “pROC” packages in R.

### 2.3 Functional and pathway enrichment analyses

Differentially expressed genes (DEGs) between groups were analyzed using “limma” (R package). Gene Ontology (GO) and Kyoto Encyclopedia of Genes and Genomes (KEGG) analyses were conducted using the KEGG Orthology Based Annotation System (KOBAS, http://bioinfo.org/kobas) database and visualized via “Goplot” and “ggplot2” (R packages). Gene Set Enrichment Analysis (GSEA) was performed to determine potential pathways using “clusterProfiler” (R package) and visualized using “ggplot” and “enrichplot” (R packages). In addition, a single GSEA of miRNA target genes was analyzed using the “GSVA” package in R via “c2.cp.reactome.v2023.1.Hs.symbols.gmt” (https://www.gsea-msigdb.org/gsea/index.jsp).

### 2.4 Tumor immune microenvironment characteristics and drug response prediction

Differences between the TMEs of high- and low-risk patients were explored by comparing GEPs in “immune_response.gmt” using the “GSVA” (Hanzelmann et al., 2013) package in R (https://www.gsea-msigdb.org/gsea/index.jsp); these were visualized using “ComplexHeatmap” (R package). Immune cell infiltration was estimated using multiple algorithms based on the GEPs, including cell-type identification by estimating the relative subsets of RNA transcripts (CIBERSORT) (Newman et al., 2015), Microenvironment Cell Populations counter (MCPcounter) (Becht et al., 2016), European Prospective Investigation into Cancer and Nutrition (EPIC) (Racle and Gfeller, 2020), and ssGSEA (Charoentong et al., 2017), and “GSVA” (R package). Pro- and anti-inflammatory cytokine ratios of the subgroups were also compared based on the average expression levels of marker genes (Li et al., 2019).

Responses to various therapeutic drugs were predicted using “oncoPredict” (R package) based on the Genomics of Drug Sensitivity in Cancer (http://www.cancerrxgene.org) database. Correlations between lncRNAs and specific drugs were analyzed using information from the LncMAP database (http://bio-bigdata.hrbmu.edu.cn/LncMAP/) and visualized using Cytoscape (version 3.9.0, http://www.cytoscape.org/23).

### 2.5 Cell culture

The HEK293T cell line (293T), and OC cell lines (CAOV3, and HEY) were purchased from Meisen CTCC (Zhejiang Meisen Cell Technology Co., Ltd., Hangzhou, China). All cell lines were cultured in Dulbecco’s modified Eagle’s medium (Gibco, Thermo Fisher Scientific Inc., Waltham, MA, United States), enriched with 10% fetal bovine serum (epizyme, Shanghai, China), at 37°C and 5% CO_2_. 

### 2.6 Generation of *RP11-508M8.1-*overexpressing cell line

We designed and synthesized the full sequence of *RP11-508M8.1 in vitro* and cloned it into the pCDH-EF1-MCS-IRES-puro vector. LncRNA-overexpressing lentivirus vectors and corresponding negative control lentiviruses were generated by packaging in 293T cells, and the viral particles were harvested after 60 h. OC cell lines were infected with the lentivirus. HEY and CAOV3 cells in good condition were selected, counted, and cultured in 10 cm cell culture dishes at 37°C and 5% CO_2_ overnight. The medium was discarded the following day, and 2 mL lentivirus and 2 mL complete culture medium were added to each dish. Infection was terminated after 18 h, and the medium was replaced with complete culture medium. After 48 h of virus addition, 0.5 mg/mL puromycin was used for screening.

### 2.7 Detection of candidate lncRNAs via reverse transcription-quantitative polymerase chain reaction (RT-qPCR)

For RNA purification, cells were lysed in TRIzol reagent (Invitrogen Life Technologies, Grand Island, NY, United States). RNA was extracted from each sample using the RNeasy Mini kit (Qiagen, Hilden, Germany). The extracted RNA was further digested with DNase I (Invitrogen, Waltham, MA, United States) to remove residual DNA. Total extracted RNA was stored at −80°C until use.

RT-qPCR was performed using a QuantStudio 6 Real-Time PCR instrument (Thermo Fisher Scientific Inc.); the reaction mixture comprised 1 µL diluted cDNA, 18.2 µL of 1 × SYBR Green PCR Master Mix, and 0.4 µL each of the forward and reverse primers (10 µmol). The PCR amplification conditions were as follows: 95°C for 5 min, followed by 40 cycles each at 95°C for 10 s and 60°C for 30 s. All samples were tested in triplicate. The relative levels of lncRNAs in cells was calculated using the following equation:

$$\text{Amount of target}=2^{-\Delta \text{Ct}},\text{where }\Delta \text{Ct}={\text{Ct}}_{lncRNA}–{\text{Ct}}_{GAPDH}$$

Gene-specific primers for lncRNA and the housekeeping gene *GAPDH* are listed in Supplementary Table S2. Three primers were designed for the RP11-508M8.1 sequence to identify the overexpression of lncRNA.

### 2.8 EdU staining assay

The proliferation ability of *RP11-508M8.1* in OC cells was determined using an EdU assay kit (Cell Light EdU DNA imaging Kit, RiboBio). A total of 1 × 10^4^ cells were seeded in 96-well plates, incubated overnight, and treated with EdU (50 μMol) for 2 h. Subsequently, the plate was removed, and the remainder of the experiment was conducted according to the instructions provided with the kit. Finally, five visual fields were randomly selected under a fluorescence microscope to acquire images as well as to calculate the proportion of EdU-positive cells.

### 2.9 Migration and invasion Transwell assays

Cell migration and invasion experiments were performed using a Transwell chamber (3422, Corning, United States). The invasion experiment required the addition of Matrigel (BD Pharmingen, San Jose, CA, United States) to the bottom of the chamber in advance, followed by a subsequent experiment post-solidification. A total of 2 × 10^5^ cells were suspended in serum-free medium in the upper chamber. For the migration assay, 1 × 10^5^ cells were suspended in serum-free medium in the upper chamber, followed by the addition of 600 μL complete medium to each culture hole. The Transwell chamber was placed in the plate and returned to the incubator for further culture for 6 (HEY) or 8 h (CAOV3). After removing the chamber, the cells were fixed with 4% paraformaldehyde for 10 min and stained with 0.1% crystal purple for 10 min. The operational procedure for the invasion experiment was consistent with that for the migration assay. Each chamber was photographed under a microscope (Leica, London, United Kingdom).

### 2.10 Wound healing assay

When the cells seeded in six-well plates reached 100% confluence, the plates were removed. Scratches were made on each plate, and the cells were rinsed gently with phosphate-buffered saline to remove floating cells. Cell culture was continued with a medium containing 2% fetal bovine serum. Representative images of cells at 0 and 24 h were obtained using a microscope, and the confluence of cells was calculated using ImageJ 1.53a (Schneider et al., 2012) to observe the invasion and migration abilities of *RP11-508M8.1* in OC cell lines.

### 2.11 Cell transfection and Western blotting

Small interfering RNA (siRNA) against METTL3 was synthesized by RiboBio (Guangzhou, China; Supplementary Table S3) and then transfected using Lipofectamine 2000 (Invitrogen Life Technologies, Carlsbad, CA, United States) according to the manufacturer’s instructions. After 48 h of transfection, cells were lysed in RIPA lysis buffer supplemented with a proteasome inhibitor. Following whole cell lysis, proteins separated via SDS-PAGE (12%) were transferred onto a PVDF membrane. The membrane was then blocked with skimmed milk and incubated with specific primary and secondary antibodies (anti-METT3: huabio; anti-GAPDH: Proteintech). Finally, protein expression was visualized using a Bio-Rad ChemiDoc Touch Imaging System.

### 2.12 RNA sequencing and analysis

Cells were collected, and total cellular RNA was extracted as described above. One microgram of total RNA was used for library preparation; poly (A) mRNA was isolated using Oligo (dT) beads, and mRNA fragmentation was performed using divalent cations under high temperature. Priming was performed using Random Primers. First- and second-strands of cDNA were synthesized; then, double-stranded cDNA was purified, treated to repair both ends, and subjected to dA-tailing in a single reaction. Subsequently, T-A ligation was performed to add adaptors to both the ends. Size selection of adaptor-ligated DNA was performed using DNA Clean Beads. Each sample was amplified via PCR using P5 and P7 primers, and the PCR products were validated. Libraries with different indices were then multiplexed and loaded on an Illumina HiSeq/Illumina Novaseq/MGI2000 instrument for sequencing using the 2 × 150 paired-end (PE) configuration according to the manufacturer’s instructions.

Pass filter data in the fastq format were processed using Cutadapt (V1.9.1, phred cutoff: 20, error rate: 0.1, adapter overlap: 1 bp, min. length: 75, proportion of N: 0.1) to remove technical sequences, including adapters, PCR primers or fragments thereof, and bases of quality lower than 20, to obtain high-quality clean data. First, human GRCh38 genome sequences and annotation files of relative species were downloaded from ENSEMBL. Then, Hisat2 (v2.0.1) was used to index the reference genome sequences. Finally, clean data were aligned to the reference genome via the Hisat2 software (v2.0.1). The initial transcripts in the fasta format were converted from a known gff annotation file and indexed properly. Next, using the file as the reference gene file, gene and isoform expression levels were estimated from cleaned pair-end data via HTSeq (v0.6.1). DEGs between groups were determined using “DESeq2” (R package) based on *p* < 0.05 and foldchange ≥1.5.

### 2.13 Dot blot assay

First, total RNA was denatured at 65°C for 5 min and transferred on to a nitrocellulose membrane (Millipore, United States) according to experimental requirements. Next, the membrane was cross-linked using UV for 30 min and washed in Phosphate Buffered Saline with Tween at room temperature for 10 min to remove the unbound RNA and subsequently sealed with milk at room temperature for 1 h. Finally, the membrane was incubated overnight with an m^6^A antibody (1:1,000, Synaptic Systems, Germany) at 4°C and a horseradish peroxidase-conjugated secondary antibody (1:1,000, Cell Signaling Technology, United States) at room temperature for 1 h. After washing, the signal of the membrane was detected using a chemiluminescence system (Bio-Rad). The membrane was stained with 0.02% methylene blue (MB) dissolved in 0.3 M sodium acetate solution (pH 5.2), and images were acquired.

### 2.14 m^6^A RNA immunoprecipitation-qRT-PCR (m^6^A MeRIP-qRT-PCR)

*METTL3* expression in ovarian cancer cells was knocked down using siRNA (Supplementary Table S3), and an m^6^A-modified RNA enrichment analysis was performed on the control and *METTL3*-knockdown cell samples according to the instructions of the riboMeRIP m^6^A Transcriptome Profiling Kit (C11051-1, RiboBio China). Briefly, 50 μg total RNA was extracted and segmented into 100–150 nt fragments, and magnetic beads with anti-m^6^A were prepared using 1/10 segmented RNA as input. The remaining segmented RNA required for MeRIP reaction solution was prepared, rotated and mixed at 4°C, and incubated for 2 h. Finally, the methylated RNA bound to the m^6^A antibody was eluted and recovered. RT-qPCR was used to detect *RP11-508M8.1* expression as well as to analyze the data following normalization to the input.

### 2.15 Identification of the lncRNA-miRNA-mRNA regulatory axis

Potential target miRNAs for candidate lncRNAs were predicted using RNAhybrid (https://bibiserv.cebitec.uni-bielefeld.de/rnahybrid), which was also used to predict the secondary structures of lncRNAs. Key miRNAs associated with the candidate lncRNA *RP11-508M8.1* were further screened based on the following criteria: (i) the miRNAs were significantly associated with survival in OC according to the ONCOMIR (https://www.oncomir.org) database and (ii) miRNA seed region (5′-> 3′) with the 2–7 bp was strictly matched with that of lncRNA. Moreover, potential target genes of these key miRNAs were further screened according to the following criteria: (i) the miRTarBase database (https://mirtarbase.cuhk.edu.cn/∼miRTarBase/miRTarBase_2022/php/ index.php) was used to predict the target genes for the key miRNAs; (ii) the target genes were further identified by combining with the DEGs (|foldchange| > 1.5 and *p* < 0.05) in cell lines overexpressing *RP11-508M8.1*; (iii) Kaplan–Meier (KM) survival analysis was applied to filter the prognosis-related mRNAs; (iv) mRNA expression levels in patients with OC were aberrant.

### 2.16 Statistical analysis

Continuous variables were analyzed using Student’s *t*-test or the nonparametric Wilcoxon test. Prognostic analyses were performed using KM survival and univariate Cox analyses. Data were analyzed using R 4.0.1 (http://www.r-project.org/). *p-*values < 0.05 were considered statistically significant.

## 3 Results

### 3.1 Construction of the m^6^A-LRM for patients with SOC

**FIGURE 1:**
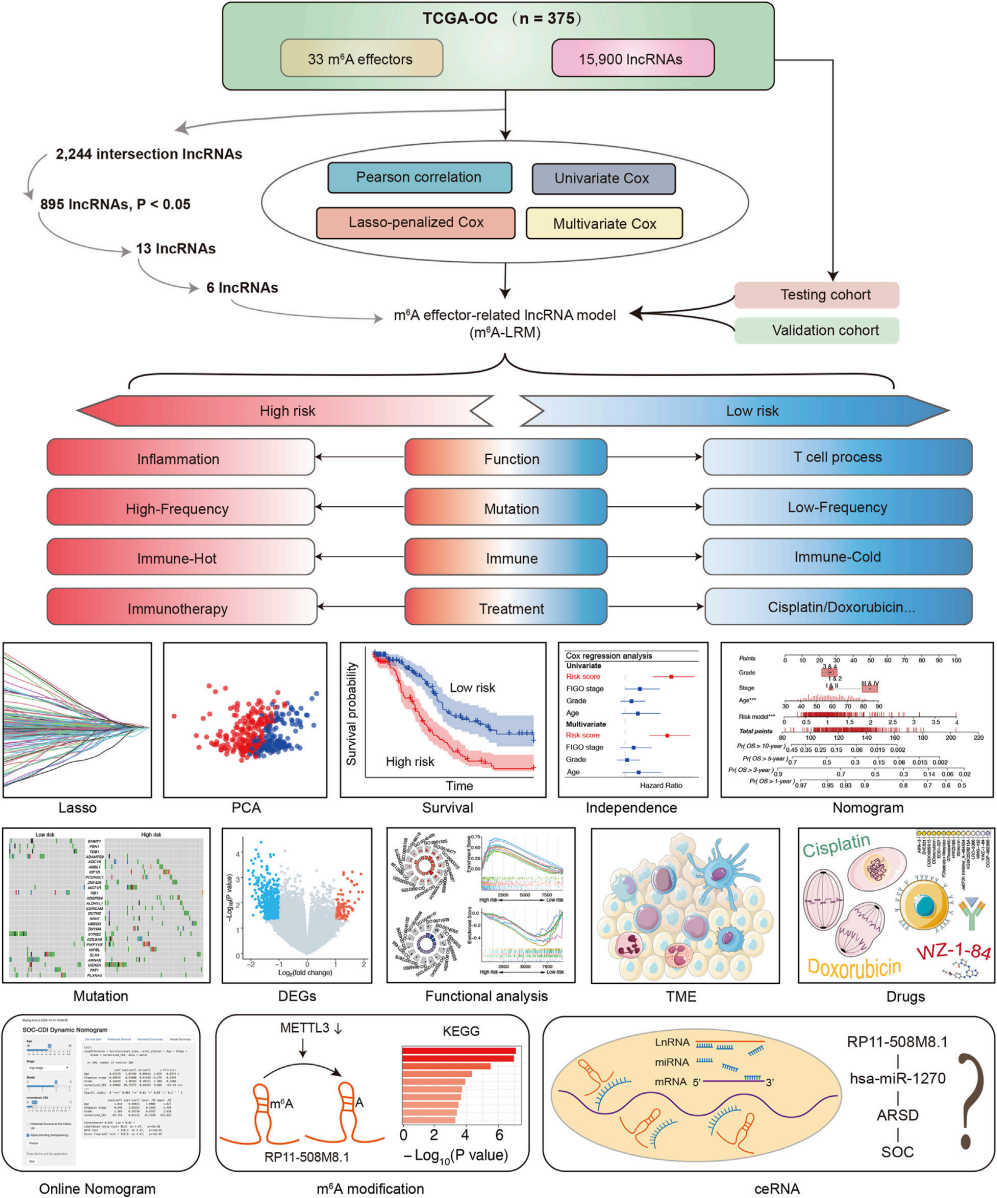
Schematic of the study.

**FIGURE 2:**
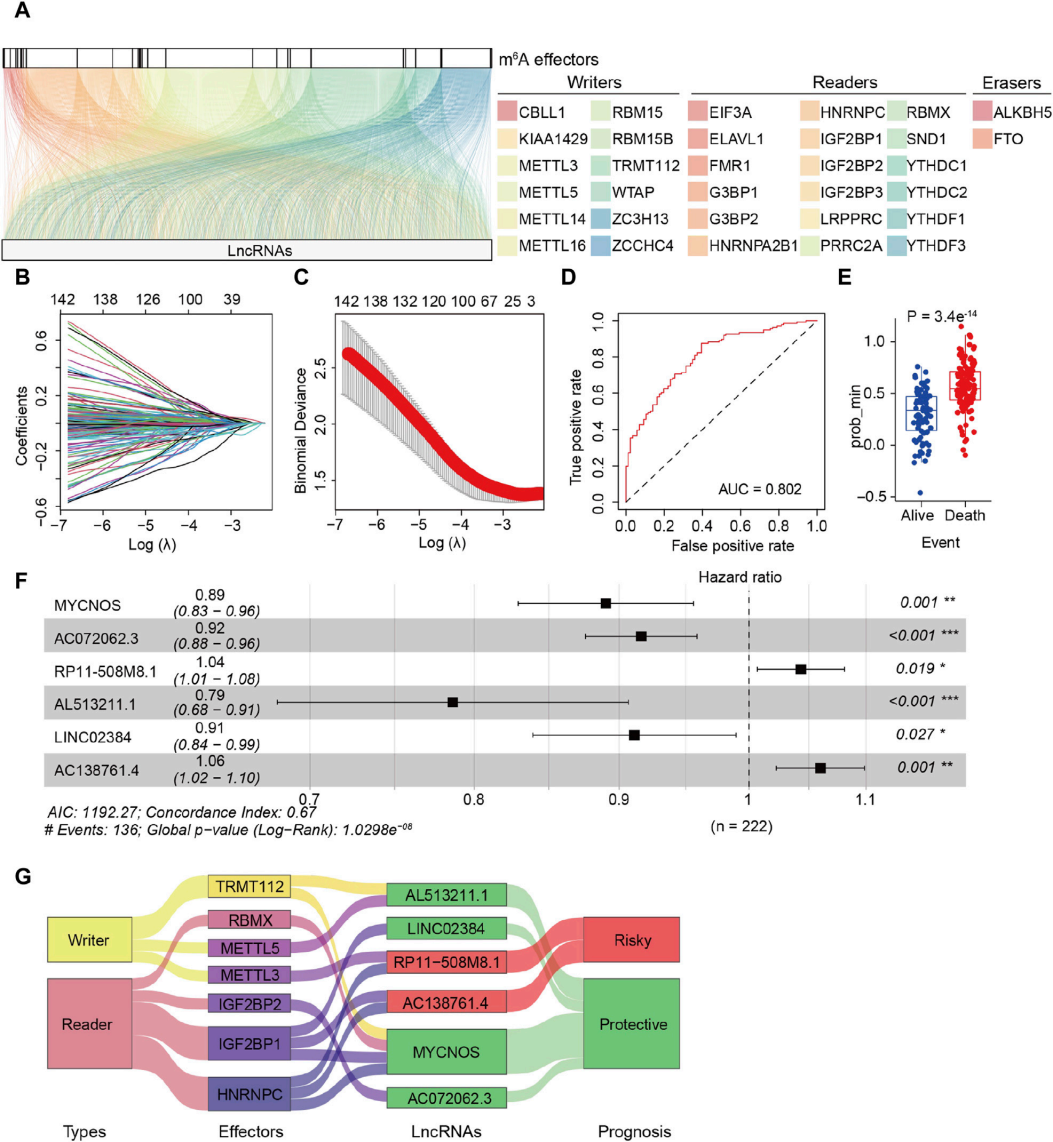
Identification of m^6^A effector-related lncRNAs in patients with serous ovarian carcinoma (SOC). **(A)** Relationship between 33 m^6^A effectors and related lncRNAs (YTHDF2 is not shown because there was no significant relationship between YTHDF2 and lncRNAs). **(B)** LASSO coefficient plots of overall survival (OS)-related lncRNAs. Perpendicular imaginary lines were drawn at the value chosen via 10-fold cross-validation. **(C)** Tuning parameters (log λ) of OS-related lncRNAs were selected to cross-verify the error curve. According to the minimal criterion (left vertical line) and 1-se criterion (right vertical line), perpendicular imaginary lines were drawn at the optimal value. **(D)** ROC curves of the model in internal validation. **(E)** Predictive discrimination of the model based on the results from minimal criterion (left panel) and 1-se criterion (right panel). **(F)** Multivariate cox regression analysis of six independent lncRNAs associated with prognosis; two were risk factors and the other four were protective factors. **(G)** Sankey diagram for correlations between 33 m^6^A effectors and 6 prognostic m^6^A effector-related lncRNAs; the diagram shows that IGF2BP1 and HNRNPC are correlated to risk-related and protective lncRNAs. In addition, different effectors were related to the same lncRNAs (METTL3/HNRNPC to RP11-508M8.1, TRMT112/RBMX/IGF2BP1/HNRNPC to MYCNOS).

The detailed procedure for identifying m^6^A effector-related lncRNAs is illustrated in Figure 1. GEP data for 33 m^6^A effectors and 15,900 lncRNAs of patients with SOC were obtained from TCGA. A total of 2,244 m^6^A effector-related lncRNAs were identified based on Pearson’s correlation analysis (|R| > 0.3 and *p* < 0.001). The m^6^A effectors and their related lncRNAs were visualized in a correlation network (Figure 2A). Among the 2,244 m^6^A effector-related lncRNAs in the training set, 895 lncRNAs that were significantly correlated with OS were identified using univariate Cox regression analysis (*p* < 0.05; Supplementary Table S4). Subsequently, we performed LASSO Cox regression analysis to identify candidate lncRNAs associated with the prognosis of patients with SOC. As a result, 13 m^6^A effector-related lncRNAs were selected based on the λ minimization method (Figures 2B, C). Model self-rating indicated that these 13 lncRNAs had significant diagnostic value (AUC = 0.802) as well as discriminatory power in the training set (Figures 2D, E). Multivariate Cox regression analysis, which was performed to control confounding factors, detected six m^6^A effector-related lncRNAs that were independently correlated with OS. Among them, RP11-508M8.1 and AC138761.4 were identified as risk factors [hazard ratio (HR) > 1, *p* < 0.05], whereas AL513211.1, LINC02384, MYCNOS, and AC072062.3 were identified as protective factors (HR < 1, *p* < 0.05; Figure 2F; Supplementary Figure S1).

**FIGURE 3:**
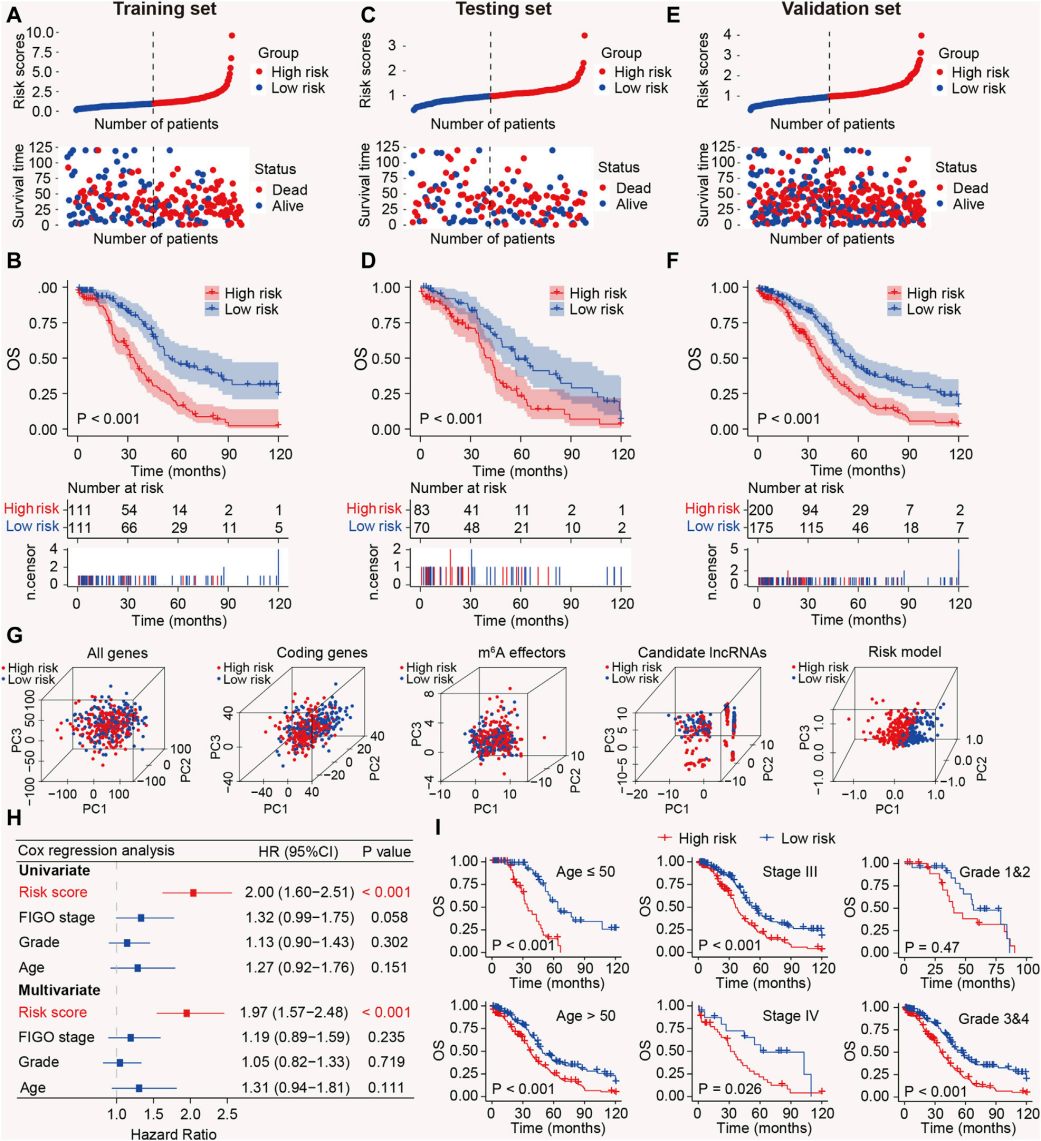
Prognostic value of the m^6^A-LRM in training, testing, and validation sets. **(A)** Distribution of m^6^A-LRM-based risk scores (upper panel), survival status, and survival time between high- and low-risk patients (bottom panel). Blue represents the low-risk group, whereas red represents the high-risk group. **(B)** KM analysis of survival of patients in the training set in the high- and low-risk groups. **(C,E)** Distribution of risk scores, survival status, and survival time of patients divided by m^6^A-LRM in the **(C)** testing set and **(E)** validation set. **(D,F)** KM survival analysis in the **(D)** testing and **(F)** validation sets. **(G)** Principal components analysis between the high- and low-risk groups based on following data: (1) All gene expression profiles, (2) Expression profiles of coding genes, (3) Expression profiles of 33 m^6^A effectors, (4), Expression profiles of six m^6^A effector-related lncRNAs, (5) m^6^A-LRM. **(H)** Univariate and multivariate analyses of clinical features and risk scores with OS; CI, confidence interval. **(I)** Prognostic ability of the risk score in distinguishing between the OS of patients ≤50 years of age and those aged >50 years (left panel). Prognostic ability of the risk score to distinguish between the OS of patients with SOC with stage III and stage IV (Middle panel). Prognostic ability of the risk score to distinguish between the OS of SOC patients with grades 1 and 2 or grades 3 and 4 (Right panel).

Subsequently, the m^6^A-LRM was constructed based on the above-mentioned six lncRNAs, the GEPs and regression coefficients of which were used to calculate prognostic risk scores in the training set. The concordance index of the m^6^A-LRM was 0.672 ± 0.025 (Figure 2F), indicating a favorable prognostic value. Surprisingly, the correlations between m^6^A effectors and candidate lncRNAs were complex, suggesting interactions and a crosstalk (Figure 2G). Patients with SOC were stratified into low- and high-risk groups based on the risk scores. The distribution of risk scores from the m^6^A-LRM and survival status of patients in the training set are shown in Figure 3A. High-risk patients had significantly shorter OS than low-risk patients (*p* < 0.001, Figure 3B).

### 3.2 External validation of the prognostic model m^6^A-LRM

To validate the prognostic ability of the m^6^A-LRM, risk scores in the testing and validation sets were determined. The distributions of risk scores, survival status, and survival time of patients with SOC are depicted (Figures 3C, E). As expected, the high-risk patients with SOC had shorter OS than the low-risk patients (*P*_testing set_ < 0.001, *P*_validation_ _set_ < 0.001; Figures 3D, F). Furthermore, the AUC values for 1-, 3-, 5-, 10-year OS estimated using the m^6^A-LRM were stable over time (Supplementary Figure S2). Furthermore, principal components analysis was performed to analyze the discriminatory power of the m^6^A-LRM for low- and high-risk patients with SOC using GEPs obtained from the following: all RNA-seq data, coding genes, 33 m^6^A effectors, 6 m^6^A effector-related lncRNAs, and m^6^A-LRM. These GEPs did not effectively discriminate between patients with SOC in the low- and high-risk groups, except for the m^6^A-LRM (Figure 3G). Interestingly, the m^6^A-LRM showed remarkable discriminatory power and provided an efficient prognostic signature in patients with SOC.

To evaluate whether the m^6^A-LRM shows potential as an independent prognosis estimator for patients with SOC, univariate and multivariate Cox regression analyses were conducted on the m^6^A-LRM risk score, the patients’ International Federation of Gynecology and Obstetrics (FIGO) stage, tumor grade, and age. Only the m^6^A-LRM risk score was found to be an independent prognostic risk factor for patients with SOC (*p* < 0.001; Figure 3H). Univariate Cox regression analysis revealed that the m^6^A-LRM risk score had HR and 95% confidence interval (CI) values of 2.00 and 1.60–2.51, respectively, similar to those obtained using the multivariate Cox regression analysis (1.97 and 1.57–2.48, respectively). These results highlighted the m^6^A-LRM risk score as the key independent prognostic factor for patients with SOC. Moreover, based on their clinicopathological characteristics, patients were stratified into low- and high-risk groups in the validation set. According to classification by patients’ age, FIGO stage, and grade, the OS of low-risk patients was longer than that of high-risk patients (Figure 3I).

### 3.3 Nomogram construction and evaluation

**FIGURE 4:**
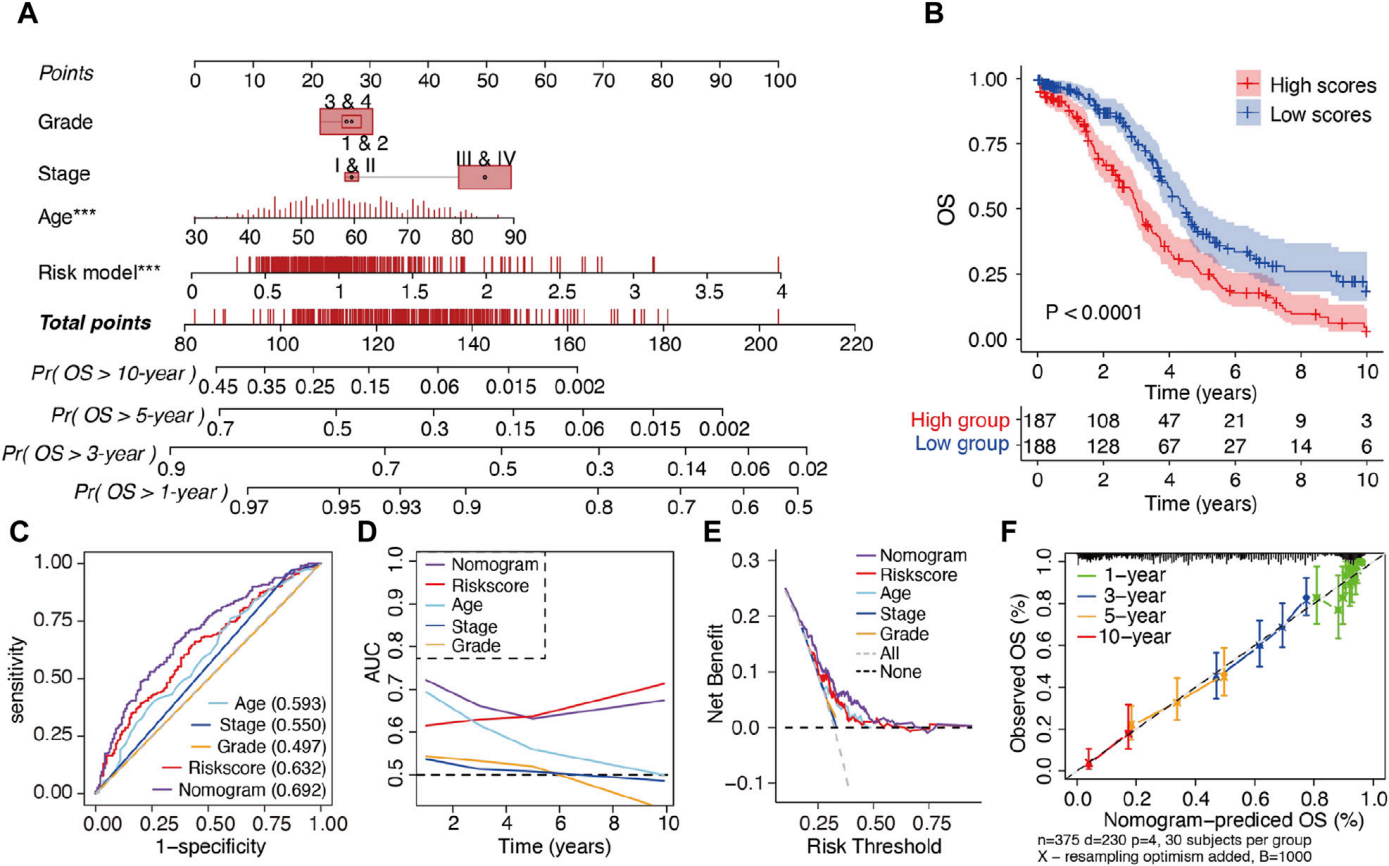
Construction and assessment of a prognostic nomogram. **(A)** Nomogram constructed using the m^6^A-LRM risk score and clinical features for 1-, 3-, 5-, and 10-year overall survival (OS). **(B)** KM survival analysis of patients in the high- and low-nomogram scores groups. **(C)** ROC curves of clinical features, m^6^A-LRM, and nomogram for OS. **(D)** Time-dependent AUC of the nomogram, m^6^A-LRM, and clinical features. **(E)** Decision curve analyses (DCAs) of the nomogram, m^6^A-LRM, and clinical features. **(F)** Calibration plot of the nomogram, showing the correlation between the actual and predicted 1-, 3-, 5-, and 10-year OS in SOC.

To enhance the clinical applicability of the m^6^A-LRM, a nomogram consisting of the m^6^A-LRM risk score, FIGO stage, tumor grade, and age of patients was constructed for predicting the 1-, 3-, 5- and 10-year OS in SOC (Figure 4A). Stratification of patients into low- and high-risk groups, based on their nomogram scores, indicated that the OS of patients with low nomogram scores was longer than that of patients with high nomogram scores (Figure 4B). Additionally, the nomogram (0.692) as well as the m^6^A-LRM (0.632) had higher ROC values than those of the other clinicopathological characteristics (Figure 4C). Moreover, the AUC value of the nomogram was greater than that of other clinical features and similar to that corresponding to m^6^A-LRM over time (Figure 4D). Compared with clinical characteristics alone, the nomogram showed a predominant predictive ability for SOC (Figure 4E). The calibration charts further displayed that the 1-, 3-, 5- and 10-year survival curves were ideally consistent between the actual and predicted OS (Figure 4F), confirming its prognostic value. Moreover, we established a user-friendly web link for clinicians (https://leley.shinyapps.io/OC_m6A_lnc/). These results suggest that the nomogram can be effectively used to assess the prognosis of patients with SOC.

### 3.4 Functional enrichment analysis of the m^6^A-related lncRNAs between low- and high-risk patients with SOC

**FIGURE 5:**
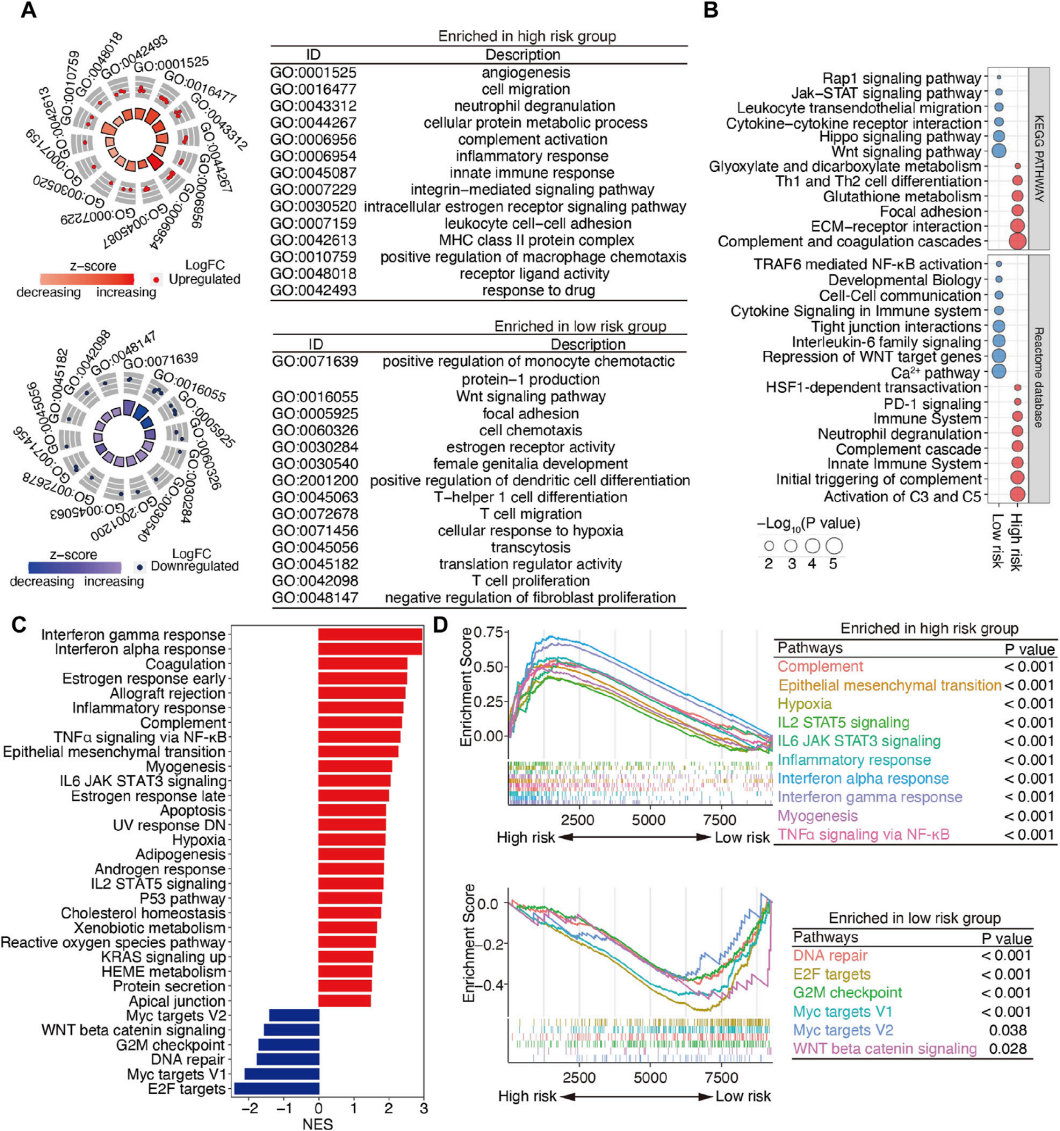
Functional enrichment analysis of m^6^A-related lncRNAs between the low- and high-risk patients with SOC. **(A)** GO terms are displayed by GOCircle plots. Red and blue dots represent the genes upregulated in the high-risk and low-risk groups separately. **(B)** Pathway analyses based on the KEGG and Reactome databases. **(C)** GSEA enrichment analysis. **(D)** GSEA plots for the two subgroups of patients with SOC. Top panel, pathways enriched the high-risk group; bottom panel, pathways enriched in the low-risk group.

To explore the underlying molecular mechanisms of m^6^A-related lncRNAs, GO, pathway, GSEA, and GSVA analyses were performed. DEGs were identified based on fold change >1.5 and *p* < 0.001. GO analysis revealed that the most significantly altered pathways in the high-risk subgroup were those mainly associated with angiogenesis, cell migration, neutrophil degranulation, innate immune response, the integrin-mediated signaling pathway, and the MHC class II protein complex. T-cell-related pathways, including T-helper 1 cell differentiation, T-cell migration, and T-cell proliferation, positive regulation of monocyte chemotactic protein-1 production, the Wnt signaling pathway, and negative regulation of fibroblast proliferation, were mainly converged in the low-risk group (Figure 5A). Pathway analyses based on two databases confirmed these findings and showed some extent of overlap with the GO analysis results (Figure 5B). A GSEA, conducted to clarify the specific roles of these pathways according to the risk categories, revealed that DEGs were enriched in inflammation-related pathways, including the interferon-gamma response, interferon-alpha response, inflammatory response, TNFα signaling via NF-𝜅B, IL6 JAK STAT3 signaling and IL2 STAT5 signaling, the epithelial mesenchymal transition (EMT), and the hypoxia and reactive oxygen species pathway (Figure 5C).

Importantly, the upregulated genes were enriched in the EMT and inflammation-related pathways, whereas the downregulated genes were enriched in DNA repair and WNT beta-catenin signaling (Figure 5D). These findings suggest that DEGs between high- and low-risk groups are implicated in the cancer–immunity pathway.

### 3.5 Characteristics of m^6^A-related lncRNAs in the tumor immune microenvironment in SOC

**FIGURE 6:**
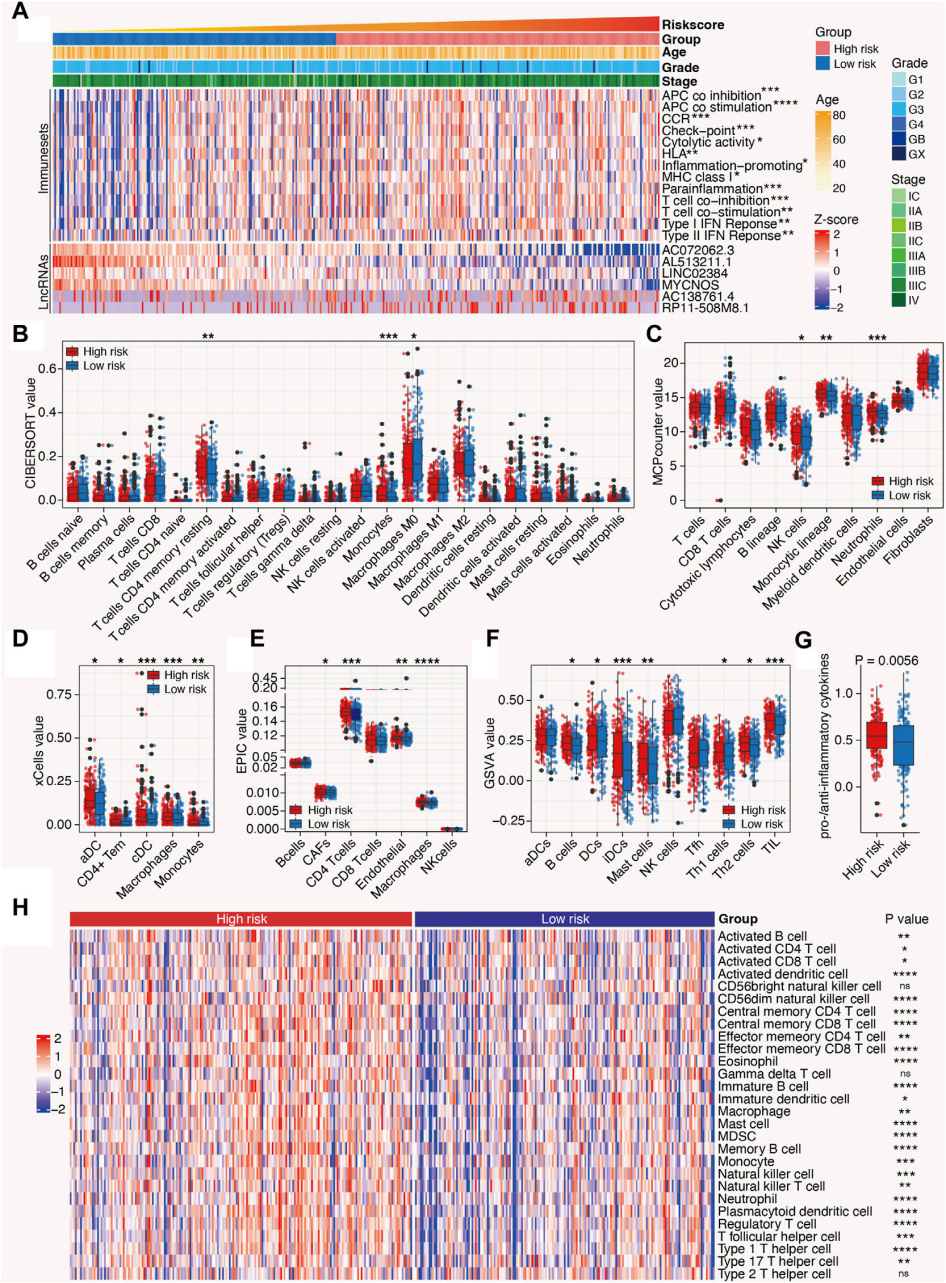
Tumor immune microenvironment characteristics of the m^6^A-related lncRNAs in SOC. **(A)** Heat map depicts the distribution of clinicopathological features and risk scores calculated by the m^6^A-LRM; value of immune sets and expression levels of lncRNAs included in the m^6^A-LRM. **(B–F,H)** Deconvolution algorithms of the CIBERSORT **(B)** and EPIC **(E)** algorithms based on the expression levels of marker genes, including MCPcounter **(C)**, xCell **(D)**, GSVA **(F)**, and ssGSEA **(H)**, were applied to estimate the immune cell infiltration status between the high- and low-risk groups. ****p < 0.0001; ***p < 0.001; **p < 0.01; *p < 0.05. **(G)** Ratios of pro-to anti-inflammatory cytokines.

Owing to the close relationship between m^6^A-related lncRNAs and the immune process, the differences between the immunological data and tumor-infiltrating immune cells associated with high- and low-risk SOC were compared. The high-risk patients with SOC had higher scores for immune sets than the low-risk patients (Figure 6A). Multiple algorithms, including CIBERSORT, MCPcounter, xCell, EPIC, and GSVA, were used to evaluate the extent of infiltration of immune cells. The expression levels of CD4^+^ T cells, monocytes, dendritic cells, B cells, Th1 cells, Th2 cells, and Tumor-infiltrating lymphocytes (TILs) in the high-risk subgroup were higher than those in the low-risk subgroup. In addition, the ratio of pro-to anti-inflammatory cytokines in the high-risk subgroup was elevated compared with that in the low-risk group (*p* < 0.05; Figures 6B–G). ssGSEA algorithms for approximately 28 immune cells were also used to substantiate the above-mentioned findings. Consistent with these results, the heat maps showed that most immune cells were enriched in the high-risk group, indicating a proinflammatory status in the high-risk group and an immune-inhibiting environment in the low-risk group (Figure 6H). These results indicate that the high-risk group is characterized by an activated immune phenotype, whereas the low-risk group exhibits a suppressed immune phenotype.

### 3.6 Mutational landscape of m^6^A-related lncRNAs in SOC

**FIGURE 7:**
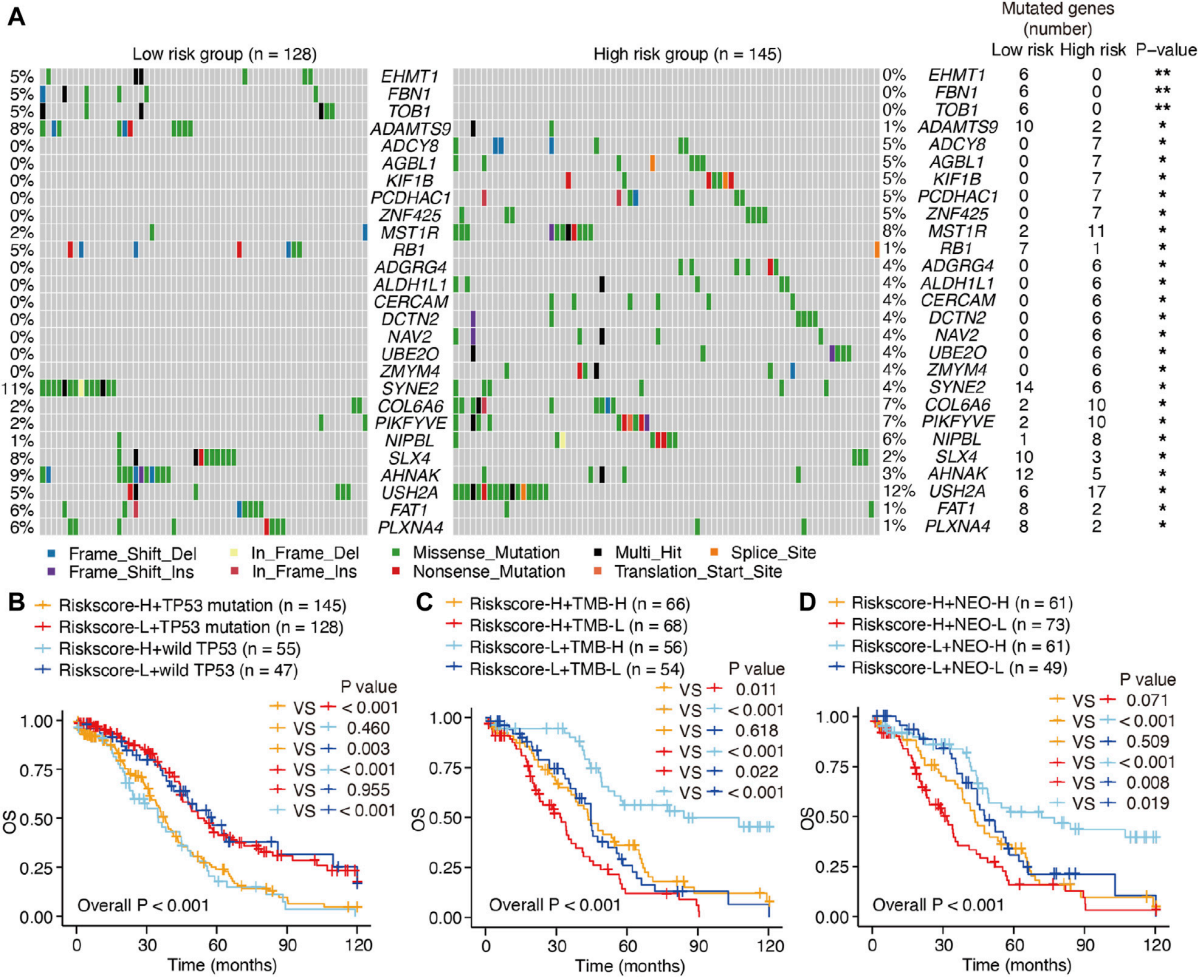
Mutations and survival in low- and high-risk patients with SOC. **(A)** Waterfall plot of differentiated somatic mutation features between the high- and low-risk groups. **(B–D)** KM survival analysis of patients classified via m^6^A-LRM risk scores, combining *TP53* mutation status, TMB loads, and neoantigens levels. TMB, tumor mutational burden; NEO, neoantigen; H, high; L, low.

Considering that hot tumors are more susceptible to immune therapy, we anticipated that patients with SOC in the high-risk group (as defined by the m^6^A-LRM) may respond to immune therapies more readily than those in the low-risk group. Previous studies have indicated that high levels of somatic mutations and neoantigens may signify a greater probability of a favorable chemotherapeutic response. We investigated the variability observed between the mutation statuses of these two groups. First, the top 20 genes with high mutation frequencies in low- and high-risk patients with SOC were identified and compared. A higher mutational rate of *USH2A* was observed in the high-risk group, while a higher mutational rate of *SYNE2* was observed in the low-risk group, with the other genes not showing any statistically significant differences (Supplementary Figure S3A). Next, we identified differentially mutated genes and found generally greater mutational rates in the high-risk group, indicating that the m^6^A-LRM did not affect frequently mutated genes but instead exerted an additive effect on those with low-frequency mutations (Figure 7A; Supplementary Figure S3A). Moreover, *TP53* had the highest mutation frequency in patients with SOC (89% and 92% in the low- and high-risk groups with gene mutation, respectively). However, no significant differences were observed in the tumor mutational burden (TMB), *TP53* mutations, and neoantigens between the low- and high-risk groups (Supplementary Figures S3A–C).

The prognostic ability of m^6^A-LRM for the TMB, *TP53* mutations, and neoantigens in patients with SOC were further explored. *TP53* effectively distinguished the survival statuses of patients with SOC (Riskscore-H + *TP53* mutation vs. Riskscore-H + wild *TP53*, *p* = 0.460; Riskscore-L + *TP53* mutation vs. Riskscore-L + wild *TP53*, *p* = 0.955; Figure 7B). Interestingly, the TMB also effectively distinguished between the survival statuses of patients with SOC (Riskscore-H + TMB-H vs. Riskscore-H + TMB-L, *p* = 0.011; Riskscore-L + TMB-H vs. Riskscore-L + TMB-L, *p* < 0.001; Figure 7C), as did neoantigens in patients with SOC with low-risk scores (Riskscore-L + NEO-H vs. Riskscore-L + NEO-L, *p* = 0.019, Figure 7D).

Furthermore, patients with SOC with high neoantigen levels in the high-risk group showed a propensity for higher OS compared with those with low neoantigen levels without significant differences (Riskscore-H + NEO-H vs. Riskscore-H + NEO-L, *p* = 0.071, Figure 7D). The m^6^A-LRM showed significant effectiveness for classifying patients who had the same *TP53*, TMB, and neoantigen status (Riskscore-H + *TP53* mutation vs. Riskscore-L + *TP53* mutation, *p* < 0.001; Riskscore-L + wild *TP53* vs. Riskscore-L + wild *TP53*, *p* < 0.001; Riskscore-H + TMB-H vs. Riskscore-L + TMB-H, *p* < 0.001; Riskscore-H + TMB-L vs. Riskscore-L + TMB-L, *p* = 0.022; Riskscore-H + NEO-H vs. Riskscore-L + NEO-H, *p* < 0.001; Riskscore-H + NEO-L vs. Riskscore-L + NEO-L, *p* = 0.008, Figures 7B–D), confirming the superiority of m^6^A-LRM over the currently available biomarkers. Additionally, we found that combining the risk scores with TMB and neoantigens increased the accuracy of prognosis estimation of patients with SOC (Riskscore-H + TMB-L vs. Riskscore-L + TMB-H, *p* < 0.001; Riskscore-H + NEO-L vs. Riskscore-L + NEO-H, *p* < 0.001, Figures 7C, D). These results indicated that the prognostic value of the m^6^A-LRM was superior to that of the TMB and neoantigens in patients with SOC.

### 3.7 Estimation of drug sensitivity and identification of novel compounds that target m^6^A-related lncRNAs in SOC

**FIGURE 8:**
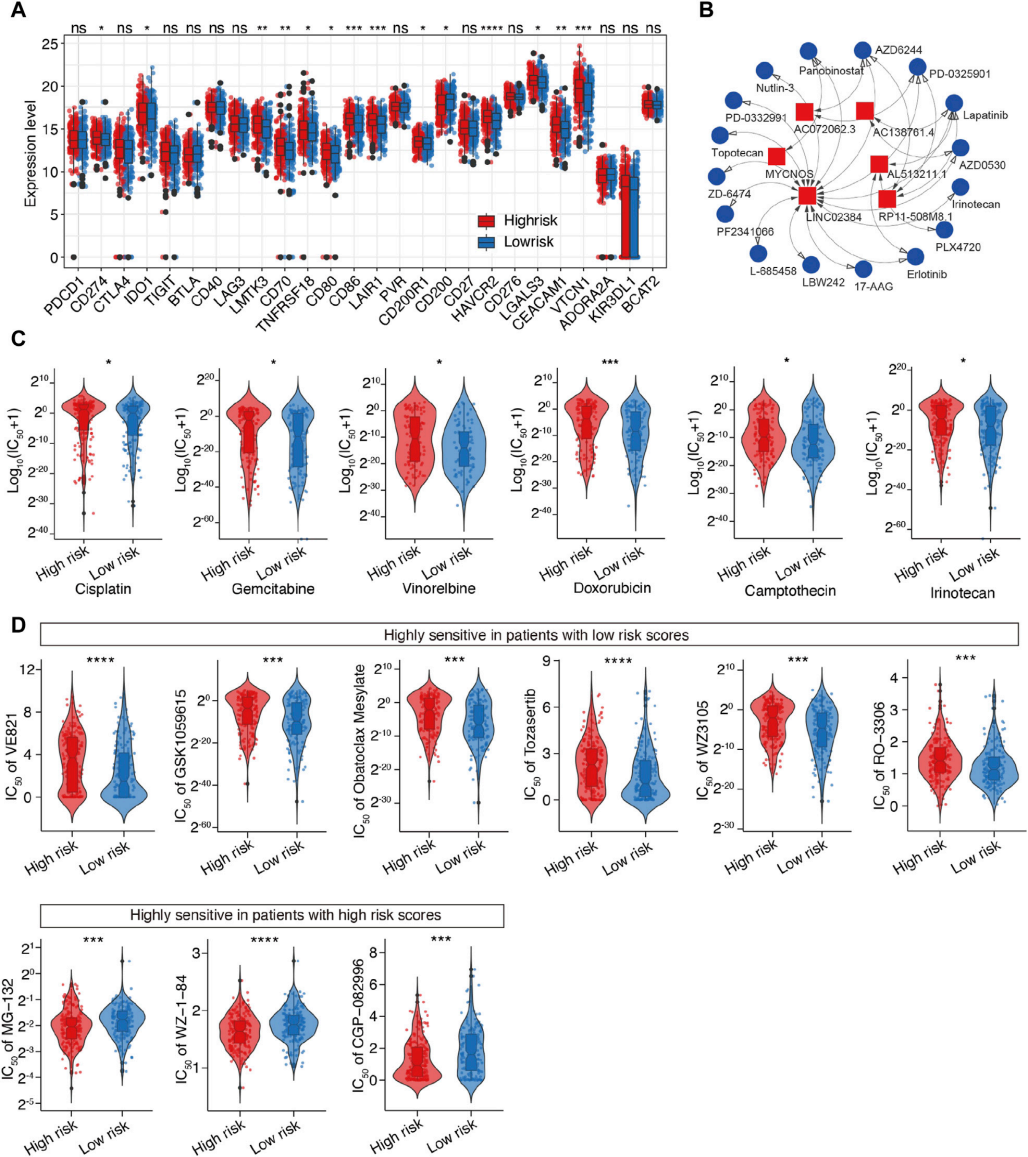
Prediction of the therapeutic response for distinct subgroups and screening of potential drugs targeting m^6^A-related lncRNAs in SOC. **(A)** Differential analysis of immune checkpoints between the two groups. **(B)** Interaction between 6 lncRNAs and 16 SOC-targeting compounds; LINC02384 exhibited the strongest correlation with compounds. **(C)** Evaluation of the sensitivities of various drug, including six common drugs for SOC. Y-axis represents the half maximal inhibitory concentration (IC_50_). **(D)** Top 10 novel candidate compounds targeting SOC according to the m^6^A-LRM (The result of Doxorubicin is shown in this figure **(C)**].

Considering the above-mentioned findings, we explored the association between m^6^A-related lncRNAs and immunotherapy. First, we compared the expression of immune checkpoints between the two subgroups. As expected, the high-risk patients were more likely to respond positively to immunotherapy than the low-risk patients and showed high expression of immune checkpoint targets, except for CD200 (Figure 8A), which suggested that risk classification based on the m^6^A-LRM may serve as an indicator for response to immunotherapy.

Next, we investigated the association between the lncRNAs utilized in the m^6^A-LRM and drug compounds to identify potential drugs targeting m^6^A effector-related lncRNAs. Interactions between lncRNAs and these drugs were predicted, resulting in the identification of 26 lncRNA-drug pairs (Supplementary Table S5); then, the complex interactions between them were observed (Figure 8B; Supplementary Figure S4). Considering its potential role in modifying immunotherapy, we used the calcPhenotype algorithm to predict the response of a common drug used for OC treatment based on the half-maximal inhibitory concentration (IC_50_) to explore the clinical use of the m^6^A-LRM. The results indicated that of the 16 commonly used drugs, six (cisplatin, gemcitabine, vinorelbine, doxorubicin, camptothecin, and irinotecan) had lower IC_50_ values (*p* < 0.05, Figure 8C) in the low-risk group. Furthermore, there was no significant difference between the IC_50_ values of other 10 drugs (Supplementary Figure S4). To further explore drugs that may potentially target SOC, we screened the Genomics of Drug Sensitivity in Cancer database. The top 10 potential compounds that exhibited significant differences in efficacy between the high- and low-risk groups are shown (Figures 8C, D). Seven compounds, including doxorubicin, displayed lower IC_50_ values in the low-risk group (Figure 8C), whereas three drugs exhibited greater sensitivity in the high-risk patients. These results indicate that the m^6^A-LRM has potential for predicting the sensitivities of certain drugs beneficial to different groups of patients with SOC.

### 3.8 Cytological function of RP11-508M8.1 in OC cells

**FIGURE 9:**
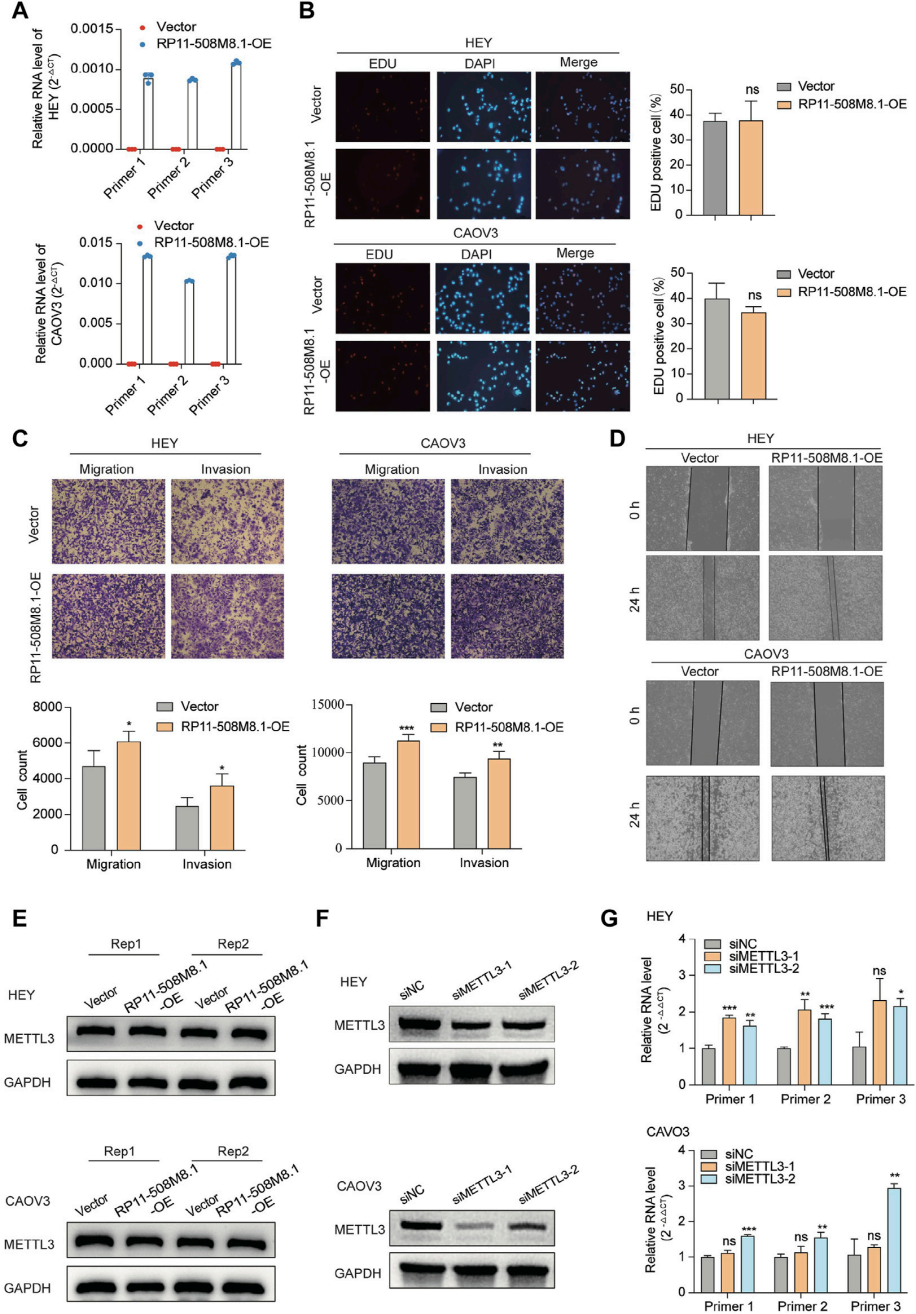
RP11-508M8.1 promotes migration and invasion of ovarian cancer cells *in vitro*. **(A)** RT-qPCR assay was used to detect the overexpression efficiency of *RP11-508M8.1* in CAOV3 and HEY OC cell lines with stable overexpression of *RP11-508M8.1* and a negative control. **(B)** Proliferation ability of HEY and CAOV3 cells overexpressing *RP11-508M8.1* was detected via an EdU assay. **(C)** Transwell representative images (upper) and quantitative results (lower) showed that overexpression of *RP11-508M8.1* enhanced the migration and invasion abilities of ovarian cancer cells. **(D)** Wound healing assay demonstrated that increased expression of *RP11-508M8.1* promoted the wound-healing ability of OC cells. Data are presented as mean ± SD. **(E)** Protein levels of METTL3 in OC cells overexpressing *RP11-508M8.1* (Rep: repeat). **(F)** Knockdown of METTL3 expression in CAVO3 and HEY cells via siRNA. **(G)** Relative RNA levels of *RP11-508M8.1* in METTL3-knockdown OC cells. *p < 0.05, **p < 0.01, ***p < 0.001.

**FIGURE 10:**
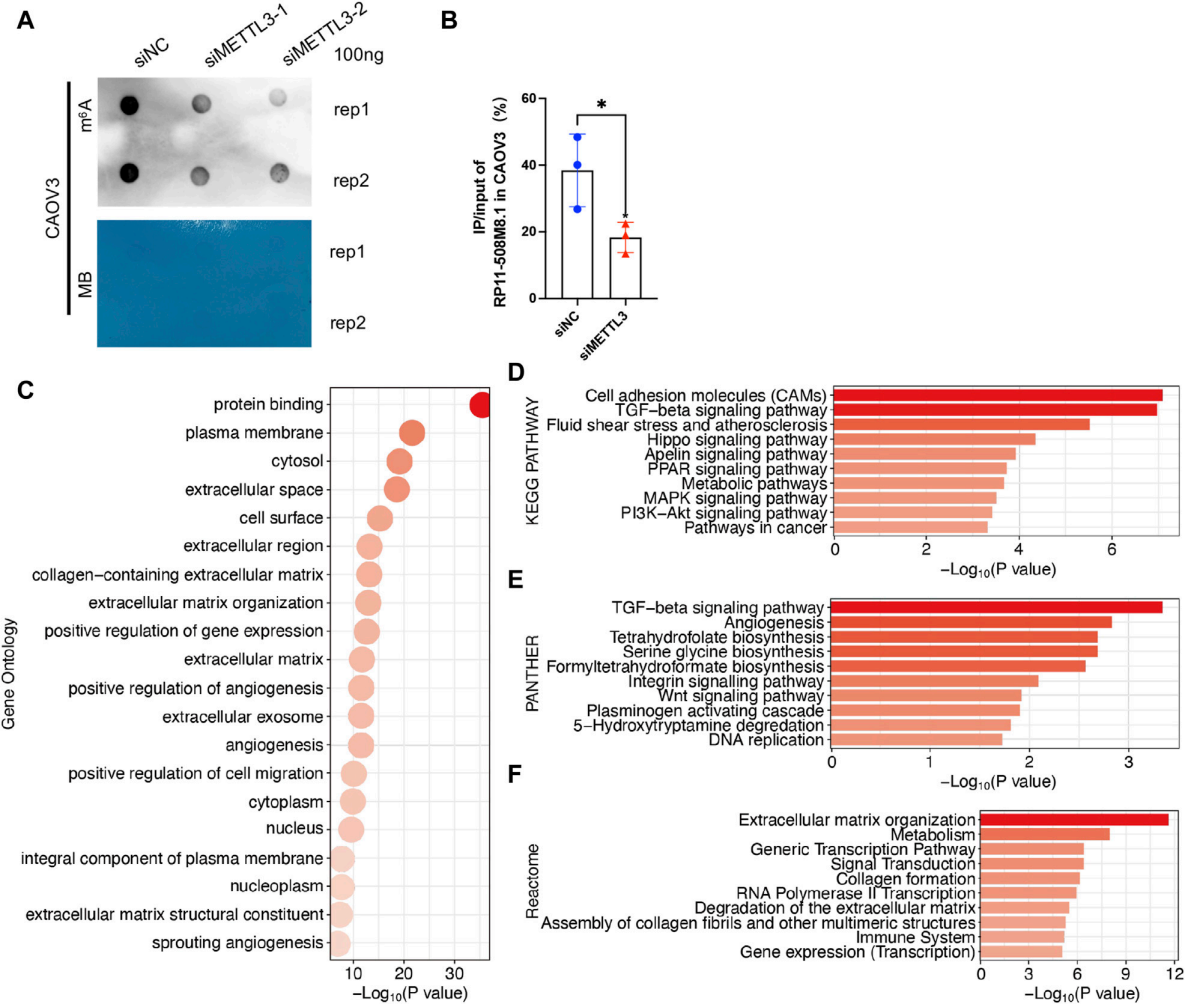
Potential function of RP11-508M8.1 in patients with SOC. **(A)** m^6^A dot blot assay in CAOV3 with knockdown of METTL3. Methylene blue (MB) stain as the loading control. **(B)** Detection of the *RP11-508M8.1* m^6^A modified levels in METTL3-knockdown CAOV3 and the normal control using MeRIP qRT-PCR. *p < 0.05. **(C)** GO analysis of DEGs in CAOV3 cells overexpressing RP11-508M8.1. **(D–F)** Pathway analyses of DEGs in CAOV3 cells overexpressing RP11-508M8.1. Based on the KEGG **(D)**, PANTHER **(E)**, and Reactome **(F)** databases.

RP11-508M8.1 is closely related to METTL3 and HNRNPC, while AC138761.4 is closely related to IGF2BP1 and HNRNPC. Previous studies have shown that METTL3 is the only catalytic subunit of the m^6^A methyltransferase complex that plays critical roles in various cancers (Deng et al., 2022; Fang et al., 2022). This information indicates that RP11-508M8.1 may play an important role in SOC. Thus, we initially selected RP11-508M8.1 and investigated its mechanism in ovarian cancer. First, in a previous study, we detected the expression of RP11-508M8.1 in normal ovaries and ovarian cancer cell lines (Ye et al., 2022). Then, to explore the functions of a candidate lncRNA, two stable SOC cell lines (HEY and CAOV3) overexpressing RP11-508M8.1 were successfully constructed (Figure 9A). Overexpression of *RP11-508M8.1* resulted in only minor effects on the proliferation of OC cell lines (Figure 9B) but significantly promoted the migration and invasion of HEY and CAOV3 (Figure 9C). Furthermore, the wound healing assay revealed that overexpression of *RP11-508M8.1* may enhance the wound-healing ability of OC cells (Figure 9D). To further explore the association between the m^6^A modification effector and lncRNA, we detected the expression of *METTL3*, which is associated with RP11-508M8.1 (Figure 2G). Overexpression of *RP11-508M8.1* did not alter the expression of *METTL3* (Figure 9E). However, the RNA expression of RP11-508M8.1 in HEY and CAOV3 was increased following *METTL3* knockdown (Figures 9F, G). These results indicated that *METTL3* is an upstream regulator of RP11-508M8.1. Furthermore, to determine the manner in which *METTL3* regulates RP11-508M8.1 expression, we conducted a dot blot assay, which revealed that *METTL3* knockdown reduced the m^6^A level of RNA in CAOV3 (Figure 10A). Next, m^6^A MeRIP-qRT-PCR was used to analyze *METTL3* expression via m^6^A-dependent regulation of *RP11-508M8.1* expression; we found that *RP11-508M8.1* was immunoprecipitated by m^6^A-MeRIP, suggesting the existence of m^6^A modification in *RP11-508M8.1*. *METTL3* knockdown significantly reduced the m^6^A enrichment level in *RP11-508M8.1* (Figure 10B). These results indicate that METTL3 may exert regulatory control over m^6^A modification, thereby modulating *RP11-508M8.1* expression.

To further explore the potential effects exerted by RP11-508M8.1 on SOC, we performed RNAseq analysis of OC cells overexpressing *RP11-508M8.1*. DEGs were identified based on fold change >1.5 and *p* < 0.001, and then GO and pathway analyses were performed. GO analysis identified the processes that were most significantly altered by RP11-508M8.1; they were mainly related to cell migration, including the plasma membrane, extracellular space, extracellular region, extracellular matrix organization, and positive regulation of cell migration (Figure 10C). Pathway analyses based on three databases confirmed these findings. DEGs were enriched in the extracellular matrix and cell movement pathways, such as cell adhesion molecules, the TGF-beta signaling pathway, angiogenesis, the Wnt signaling pathway, extracellular matrix organization, and extracellular matrix degradation (Figures 10D–F). These results indicated that RP11-508M8.1 may play an oncogenic role by affecting extracellular matrix organization and cell migration.

### 3.9 Identification of a ceRNA regulatory axis

**FIGURE 11:**
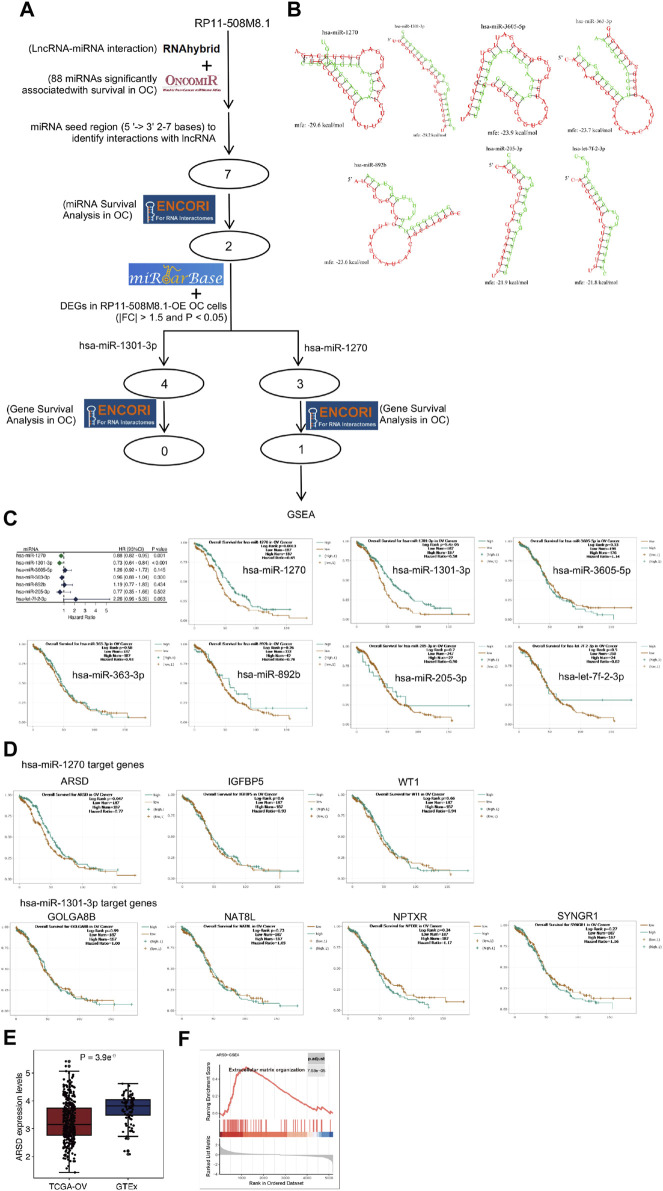
Construction of an lncRNA-miRNA-mRNA regulatory network and bioinformatic analysis. **(A)** Data analysis-based overview of the lncRNA-miRNA-mRNA regulatory axis. **(B)** Seven miRNA targets of the lncRNA PR11-508M8.1. **(C)** Univariate Cox regression and KM survival analysis of the highly connected miRNAs based on TCGA and ENCORI databases. **(D)** The correlation between survival possibility and the expression of hsa-miR-1270 targets (ARSD, IGFBP5, and WT1) and has-miR-1301-3p targets (GOLGA8B, NAT8L, NPTXR, and SYNGR1) is shown via KM analysis using data from the ENCORI database. **(E)** Expression of ARSD in SOC and normal ovary tissues. **(F)** Single gene enrichment analysis of ARSD.

RP11-508M8.1 may be involved in the progression of OC. The potential molecular mechanism underlying the role of RP11-508M8.1 in SOC was subsequently investigated using a regulation axis of ceRNA interactions (Figure 11A). The RNAhybrid and ONCOMIR databases predicted the presence of a total of seven miRNAs (hsa-miR-1270, has-miR-1301-3p, hsa-miR-3605-5p, hsa-miR-363-3p, hsa-miR-892b, hsa-miR-205-3p, and hsa-let-7f-2-3p) that bind to *RP11-508M8.1* under the following conditions: miRNA seed region (5′- > 3′) and 2–7 bp should be closely matched with the lncRNA to execute screening (Figure 11B). However, univariate Cox regression and KM analyses showed that only hsa-miR-1270 and has-miR-1301-3p expression was correlated with the survival of patients with OC (Figure 11C). Furthermore, analysis of data from the miRTarBase database indicated that the gene targets binding to the miRNAs hsa-miR-1270 and has-miR-1301-3p may constitute the miRNA-mRNA axis. We combined the DEGs in *RP11-508M8.1*-OE OC cells to filter the genes. A total of three (*ARSD, IGFBP5,* and *WT1*) and four (*GOLGA8B, NAT8L, NPTXR,* and *SYNGR1*) gene targets were found to bind hsa-miR-1270 and has-miR-1301-3p, respectively (Figure 11D). The ENCORI database, which was used to perform gene survival analysis in OC, showed that ARSD expression (hsa-miR-1270 targeted gene) alone was significantly associated with survival and that patients with higher ARSD expression had a higher survival possibility, with an HR < 1 (Figure 11D). In addition, ARSD expression was also detected; the results suggested that ARSD was downregulated in OC tissues compared to that in normal tissues (Figure 11E). Moreover, ssGSEA showed that ARSD expression was correlated with the extracellular matrix organization pathway (Top one enrichment), indicating that ARSD may serve as a regulatory factor in tumorigenesis and tumor progression (Figure 11F). These findings indicated that the RP11-508M8.1/hsa-miR-1270/ARSD regulatory axis may be of importance in the progression of OC.

## 4 Discussion

### 4.1 Potential of m^6^A-related lncRNAs as biomarkers in SOC to improve PPPM

High-grade SOC is the most prevalent and aggressive form of SOC, which is an intractable disease (Drumond-Bock and Bieniasz, 2021). Most patients with SOC are diagnosed at stage III or IV, which results in a significant reduction in their responsiveness to treatment as well as survival (Drumond-Bock and Bieniasz, 2021). Many studies have focused on identifying reliable early diagnostic biomarkers, novel therapeutic targets, and prognostic biomarkers to improve the prognosis of patients in advanced stages (van Zyl et al., 2018). However, the currently used imaging, histological evaluation, serum markers (i.e., CA125), and predictive models for managing SOC lack sensitivity and specificity, making it difficult to meet the needs of PPPM (Punzón-Jiménez et al., 2022). The m^6^A modification, which is considered the most common modification among lncRNAs (Patil et al., 2018), has currently become the focus of attention of cancer researchers. The m^6^A modification and its effectors influence the fate of RNA molecules via lncRNA regulation, often resulting in the onset and development of cancers (Dai et al., 2020; Huang et al., 2020). LncRNAs regulated by the m^6^A modification have shown potential applicability in the diagnosis, treatment, and prognosis of various cancers (Dai et al., 2020), especially as diagnostic and prognostic tools in clinical settings, thereby facilitating the prediction, targeted prevention, and personalized treatment of SOC.

Considering that the functions of lncRNAs are dynamically regulated by m^6^A writers, readers, and erasers (He et al., 2020) and that the role of lncRNAs in cancer has been attributed to integrated m^6^A effector regulation (Lan et al., 2021), the present study analyzed a comprehensive set of m^6^A effectors. In this study, we identified six m^6^A effector-related lncRNAs (RP11-508M8.1, AC138761.4, AL513211.1, LINC02384, MYCNOS, and AC072062.3) and constructed a risk model, m^6^A-LRM, to accurately predict the OS of patients with SOC as well as their response to treatment. Of these six lncRNAs, only MYCNOS and LINC02384 were extensively investigated. MYCNOS expression is associated with various cancers. For example, MYCNOS, which is upregulated in hepatocellular carcinoma cells and tissues, affects disease progression, shortens patient survival (Yu et al., 2020) and acts as an endogenous sponge of miR-216b, thereby regulating the expression of FOXM1 and promoting the proliferation of glioblastoma cells (Zhao et al., 2021). MYCNOS upregulation is associated with poor prognosis in neuroblastoma patients (Vadie et al., 2015). Interestingly, in this study, MYCNOS was identified as a protective factor in patients with SOC. However, lncRNAs reportedly play opposing roles in different cancers via crosstalks among multiple mechanisms (Fang and Fullwood, 2016; Goodall and Wickramasinghe, 2021); thus, the role of MYCNOS in SOC may require further investigation. Furthermore, studies have suggested that LINC02384, which stimulates melanoma progression by reducing the expression of the tumor-protecting miRNAs miR.891a.5p and miR.203b.3p (Zhang C. et al., 2021), may also act as a protective factor in renal cell carcinoma (Li et al., 2021) and breast cancer (Xu Z. J. et al., 2021). Although the results of the current study indicated that LINC02384 may act as a protective factor in SOC, data on the remaining four lncRNAs are lacking. The findings of subsequent univariate and multivariate analyses indicated that the m^6^A-LRM may also be useful as an independent prognostic factor for SOC. Moreover, the m^6^A-LRM may predict risks across different age groups. However, the risk model demonstrated predictive trends (*p* > 0.05) only when stratifying the OS of stage IV and grade 1 and 2 patients with SOC. This may be attributed to the limited sample size. The nomogram further indicated that risk models based on m^6^A effector-related lncRNAs exhibited a strong association with SOC and may therefore serve as a valuable tool for effective risk stratification of patients with SOC.

### 4.2 Application of the m^6^A-LRM in immunotherapy and chemotherapy

To explore the potential of the m^6^A-LRM in predicting the immunotherapeutic response of SOC, we performed comparative analyses of tumor-infiltrating immune cell levels, ICIs expression, tumor mutations, and neoantigen loads. The TME, including immune cells, cytokines, and chemokines, exhibits high heterogeneity and plasticity, which evolve with tumor progression, thus forming a complex immune landscape (Hiam-Galvez et al., 2021; Liu and Sun, 2021). Dendritic cells initiate anti-tumor immunity by capturing and presenting tumor antigens, which activate CD8^+^ and CD4^+^ T cells (Jhunjhunwala et al., 2021). However, tumor cells remodel the TME to augment immune-suppressive cells, thereby evading immune surveillance (Cao et al., 2023; de Visser and Joyce, 2023). Growing evidence suggests that m^6^A modification regulates the metabolism and activation of immune cells as well as the processes associated with immune response, thereby playing a pivotal role in reshaping the TME and orchestrating immune evasion in tumors, which in turn undermines the efficacy of immunotherapy (Li X. et al., 2022; Cao et al., 2023). LncRNAs not only play a key regulatory role in the process of proliferation, migration, and invasion of cancer cells but also act as active participants in the immune system by regulating the development, differentiation, and function of various immune cells (Chen et al., 2017; Denaro et al., 2019; Zhang Y. et al., 2021). In this study, we constructed the m^6^A-LRM using m^6^A effector-related lncRNAs and applied the model to stratify the risk of patients with SOC. Moreover, we comprehensively analyzed the disparities between the TMEs of the high- and low-risk groups to determine immune cell infiltration patterns within the TME. We found that the counts of CD4^+^ T cells, monocytes, dendritic cells, B cells, Th1 cells, Th2 cells, and TILs in the high-risk group were increased and the proportion of pro- and anti-inflammatory cytokines in the high-risk group was higher than that in the low-risk group, indicating an adaptive immune activation status. Considering the proinflammatory immune milieu observed in the high-risk group and the immunosuppression environment observed in the low-risk group, it is plausible that the high- and low-risk groups based on the m^6^A-LRM signature may encompass “hot” and “cold” tumors, respectively. Thus, the high-risk group may display elevated responsiveness toward immunotherapeutic interventions.

Cancer cells may suppress the immune system by activating immune checkpoints, a class of immunosuppressive molecules that are expressed on immune cells and regulate the extent of immune activation (Darvin et al., 2018). ICIs, which exert an oncostatic effect by enhancing T-cell activation and proliferation, are emerging as potential therapeutic modalities for cancer (Darvin et al., 2018; Ribas and Wolchok, 2018). Many ICIs, such as cytotoxic T lymphocyte associate protein-4 (CTLA-4), programmed cell death-1 (PD-1), and programmed cell death-ligand 1 (PD-L1) antibodies, have been applied in clinical settings (Postow et al., 2015). The TMB is an independent biomarker used to determine the suitability of patients for immunotherapy. A higher TMB leads to tumorigenesis and more neoantigens, which in turn drive T cell-mediated antitumor immune responses; thus, patients with high TMB may benefit more from immunotherapy than patients with low TMB (Jardim et al., 2021). In the present study, we found that low-frequency mutations that were closer to the upper end of the low frequency range were prevalent in the high-risk group, indicating that the high-risk group was more suitable for immunotherapy. We also confirmed that the established m^6^A-LRM had superior predictive power with respect to the prognosis of patients with SOC compared with that of the TMB and neoantigens. Thus, the m^6^A-LRM shows potential as a novel prognostic marker for patients with SOC. Furthermore, the expression levels of immune checkpoints may be compared to assess their effectiveness in patients receiving immunotherapy. We found that the expression levels of IC-related genes, such as *HAVCR2, CD86, LAIR1,* and *VTCN1,* in the high-risk group were significantly higher than those in the low-risk group, thereby explaining the higher sensitivity shown by the high-risk group to immunotherapy and confirming the high-risk group as a “hot tumor” group.

Models based on the m^6^A-LRM signature may also be used to predict the chemotherapy response of patients with SOC. Drug sensitivity experiments revealed that the susceptibility of the low-risk patients to conventional chemotherapeutic agents, such as cisplatin, gemcitabine, vinorelbine, doxorubicin, camptothecin, and irinotecan, was enhanced. Based on the close association between the m^6^A-LRM and immunotherapy response, potential lncRNA-targeting chemicals were identified for future exploration. AZD6244, PD-0325901, and lapatinib were the top three drugs predicted as being capable of targeting multiple candidate lncRNAs. The MEK1/2 inhibitor AZD6244 reportedly inhibited the growth of clear cell ovarian carcinoma (Bartholomeusz et al., 2012). Sheppard et al. demonstrated that PF-04691502 and PD-0325901 synergistically inhibited the growth of OC cells (Sheppard et al., 2013). Meanwhile, treatment with nanocolloids of paclitaxel and lapatinib effectively overcame the multi-drug resistance of OC cells (Vergara et al., 2012). These findings imply that the m^6^A-LRM may potentially be used to evaluate treatment response, assess prognostic risk, and develop personalized treatment strategies for individuals with SOC, thereby demonstrating a superior ability to improve PPPM in SOC.

### 4.3 Molecular mechanisms underlying the functions of m^6^A-related lncRNAs

Functional enrichment analyses and variation landscapes of the high- and low-risk groups may provide insights into the effects and underlying molecular mechanisms of m^6^A-related lncRNAs. Such experiments may help optimize the prediction model, further reveal the association between the immune microenvironment and m^6^A-related lncRNAs, provide more treatment choices, and reveal the presence of additional SOC-related pathways.

Our study showed that the high-risk group was in a state of immunophenotype activation. Such immune signatures may be explained via molecular signatures. GSEA indicated that the upregulated genes in the high-risk group were significantly enriched in the EMT and inflammation-related pathways. EMT refers to the transformation of epithelioid cells into mesenchymal phenotypic cells, which is recognized as malignant cellular behavior that facilitates tumor metastasis (Huang et al., 2022). EMT interacts with the tumor immune microenvironment in a significant manner (Dongre and Weinberg, 2019). T lymphocytes and macrophages may induce cancer cell EMT, thereby facilitating the recruitment of various immune cells, including immunosuppressive regulatory T cells, to inhibit tumor immunity and promote PD-L1 expression in cancer cells (Dongre and Weinberg, 2019). EMT may well account for the poor prognoses and proinflammatory statuses observed in the high-risk group. The genes that were downregulated in the low-risk group were enriched in the DNA repair and WNT beta-catenin signaling pathways. DNA damage repair (DDR) maintains the genome integrity of cancer cells, which plays a role in cancer progression, while downregulation of the DNA repair pathway corresponded to better prognoses in the low-risk group in our study (Xie et al., 2021). However, upregulation of DNA repair genes is linked to a lack of immune cell infiltration, which is inconsistent with the immunophenotypic suppression observed in the low-risk group (Higgs et al., 2022). The association between DDR and the immune microenvironment requires in-depth investigations, with particular reference to the treatment efficacy of DDR inhibitors combined with ICIs, which has attracted the attention of researchers (Sheng et al., 2020). The WNT beta-catenin signaling pathway is known to be associated with carcinogenicity. More importantly, the activation of WNT beta-catenin signaling is positively correlated with DDR and EMT, which jointly participate in cancer progression as well as in the shaping of the immune microenvironment (Hashemi et al., 2023). These pathways as well as a potential crosstalk between them are essential aspects of the molecular mechanism underlying the accurate prediction of tumor characteristics by the m^6^A-LRM.

In terms of the differences between the mutation landscapes of the high- and low-risk groups, *USH2A* had a higher mutation frequency in the high-risk group. A study found that ICIs exhibit better efficacy in patients carrying *USH2A* missense mutations, thereby providing an important reference for treatment selection in high-risk patients (Yang et al., 2023). These findings are consistent with those of Sun et al., who suggested that the mutation of *USH2A* was associated with an increase in the TMB and antitumor immunity (Sun et al., 2021). *SYNE2* showed a higher mutation frequency in the low-risk group. A previous study suggested that ovarian cancer cell clusters with higher mutation burden tend to display high mutation rates of SYNE2 (Li L. et al., 2022), which is inconsistent with our results. We hypothesize that this discrepancy may be attributed to variances within the analyzed cohort and grouping. Specific reasons for these conflicting results warrant further research.

### 4.4 RP11-508M8.1 regulates the expression of ARSD via hsa-miR-1270

The results of this study indicated that RP11-508M8.1 was strongly associated with the m^6^A-writer METTL3. METTL3 is a risk factor for SOC and a core m^6^A methyltransferase that plays critical roles in various cancers (Zeng et al., 2020). The function of RP11-508M8.1 in OC was explored *in vitro*. Preliminary results indicated that PR11-508M8.1 promoted OC cell invasion and migration. Although PR11-508M8.1 overexpression did not alter METTL3 levels, downregulating METTL3 increased RP11-508M8.1 expression. These findings indicate that METTL3 may be an upstream regulator of RP11-508M8.1 and that the METTL3-m^6^A-RP11-508M8.1 axis plays a role in the carcinogenicity mechanism underlying SOC.

ceRNA refers to RNA molecules such as mRNA, lncRNA, and circRNA that can competitively bind miRNAs to alter the transcriptional levels of miRNA-regulated mRNAs, thus exerting biological functions in cancer (Tay et al., 2014; Braga et al., 2020). In recent years, the ceRNA regulatory network has garnered significant attention as a novel mechanism underlying RNA interactions (Thomson and Dinger, 2016). Therefore, we investigated the ceRNA network of RP11-508M8.1 and established a novel lncRNA-miRNA-mRNA regulatory network, which has not been previously reported in relation to SOC. Our results indicated that RP11-508M8.1 may regulate ARSD expression by altering hsa-miR-1270 expression. This regulatory axis may activate protumor pathways (e.g., EMT, reactive oxygen species pathway, and extracellular matrix organization pathway). Previous research has shown that miR-1270 plays a novel tumor suppressor role in lung adenocarcinoma (Saproo et al., 2023). Hsa-miR-1270 suppresses the malignant progression of breast cancer by regulating gene expression (Hu et al., 2022). A previous study reported that ARSD exerts inhibitory effects on the proliferation and migration of breast cells by activating the Hippo/YAP pathway (Lin et al., 2021). Here, we identified ARSD as a potential protective factor in the context of SOC, exhibiting an anti-tumorigenic role. Furthermore, ARSD serves as a prognostic biomarker that facilitates the progression of glioma cells via the activation of the JAK2/STAT3 signaling pathway and infiltration of M2 macrophages (Song et al., 2023). Thus, ARSD may act as a potential novel biomarker that may improve the prognosis of patients with SOC.

### 4.5 Limitations

The current study was affected by some limitations. First, our data analysis was derived from TCGA data; thus, further large-scale investigations are required to corroborate our findings. Second, an increasing body of evidence indicates that various modification types may interact during tumorigenesis and progression, thereby establishing a complex regulatory network. Consequently, additional modifications should be incorporated into future studies to elucidate the specific molecular mechanisms underlying SOC. Third, the data used to analyze and construct the model were obtained from ovarian cancer samples, and the role played by N6-methyladenosine effector-related lncRNAs signature in other cancers remains to be explored. Fourth, the expression and biological function of RP11-508M8.1 *in vivo* must be verified in the future. Fifth, further investigation should be performed to elucidate the intricate regulatory network between m^6^A effector HNRNPC and lncRNA RP11-508M8.1. Moreover, it is necessary to screen specific mutant cell lines to further explore the potential in the pathogenesis and progression of SOC. Finally, the ability of the developed risk model to predict the response to immunotherapy was only indirectly evaluated. These findings remain to be confirmed by future studies possibly involving *in vitro* drug sensitivity tests.

## 5 Conclusion

We constructed a novel risk prediction model for patients with SOC based on six m^6^A effector-related lncRNAs, namely, RP11-508M8.1, AC138761.4, AL513211.1, LINC02384, MYCNOS, and AC072062.3. This novel risk prediction model effectively evaluated the survival rate and treatment response in relation to SOC. A free web application of the m^6^A-LRM for researchers and clinicians was developed and may provide reference information for precision treatment, thereby facilitating the PPPM of SOC. The influence of m^6^A-LRM on SOC was explored from multiple perspectives, and the association between m^6^A effectors and key lncRNAs as well as the preliminary mechanisms underlying their effect on OC were explored via *in vitro* experimentation. In conclusion, we propose that the regulatory axis involving METTL3/m^6^A/RP11-508M8.1/hsa-miR-1270/ARSD may represent one of the molecular mechanisms underlying SOC.

## Data Availability

The RNA-Seq datasets presented in this study can be found in online repositories. The names of the repository/repositories and accession number(s) can be found below: https://www.ncbi.nlm.nih.gov/, PRJNA1119078.
