# Supplementary material for: Identification of potential novel N6-methyladenosine effector-related lncRNA biomarkers for serous ovarian carcinoma: a machine learning-based exploration in the framework of 3P medicine
Source: Front Pharmacol. 2024 Jun 4;15:1351929. doi: 10.3389/fphar.2024.1351929 (PMC11185051; doi:10.3389/fphar.2024.1351929)
Supplement: Supplementary file 1 [file DataSheet1.docx]

Supplementary Material

# Supplementary Figures and Tables

**Supplementary** **Table 1. Information regarding the two subgroups of patients retrieved from TCGA database.**

|  | Training set | Testing set |
| --- | --- | --- |
|  | n = 222 | n = 153 |
| Age (years) | 59.9 (30-87) | 59 (34-87) |
| Grade |  |  |
| 1 | 1 | 0 |
| 2 | 25 | 17 |
| 3 | 189 | 132 |
| 4 | 1 | 0 |
| B | 1 | 1 |
| X | 5 | 1 |
| missing | 0 | 2 |
| Stage |  |  |
| IC | 1 |  |
| IIA | 1 | 2 |
| IIB | 2 | 1 |
| IIC | 11 | 5 |
| IIIA | 3 | 4 |
| IIIB | 9 | 4 |
| IIIC | 157 | 115 |
| IV | 36 | 21 |
| missing | 2 | 1 |

**Supplementary Table 2. Primers used for RT-qPCR.**

| lncRNA |  | Primer sequence (5′ to 3′) | Used in this study |
| --- | --- | --- | --- |
| *RP11-508M8.1*- Primer 1 | Forward | GAGGCTGAGTGAGCTGAAAG | Relative RNA expression level |
|  | Reverse | GATTGTATGTGATGGGTTGGA |  |
| *RP11-508M8.1*- Primer 2 | Forward | TGATTTCCCTGTCTGGTGCG |  |
|  | Reverse | TCAGGGGATACTGGGCTTCT |  |
| *RP11-508M8.1*- Primer 3 | Forward | TCCCGCCACATTTGAAGAAC |  |
|  | Reverse | AGCTGTCATGTTGCTGATCCA |  |
| *GAPDH* | Forward | CAGGGCTGCTTTTAACTCTGGTAA |  |
|  | Reverse | GGGTGGAATCATATTGGAACATGT |  |
| *RP11-508M8.1* | Forward | GAGGCTGAGTGAGCTGAAAG | MeRip-qPCR |
|  | Reverse | GATTGTATGTGATGGGTTGGA |  |

**Supplementary Table 3. Sequences of siRNA for METTL3.**

| siRNA | Sequences (5′ to 3′) |
| --- | --- |
| siMETTL3-1 | CAAGTATGTTCACTATGAA |
| siMETTL3-2 | GACTGCTCTTTCCTTAATA |

**Supplementary Table 4. Univariate cox regression analysis of lncRNAs with overall survival (OS) in the training set.**

| LncRNA | Hazard ratio (95% CI) | P-value |
| --- | --- | --- |
| RP11-146F11.1 | 0.45 (0.31–0.65) | 1.84E-05 |
| RP1-228H13.5 | 0.42 (0.28–0.62) | 1.92E-05 |
| RP11-140K17.3 | 0.42 (0.28– 0.63) | 1.98E-05 |
| RP5-827C21.4 | 0.33 (0.20–0.55) | 2.00E-05 |
| CTC-543D15.8 | 0.37 (0.23–0.60) | 4.90E-05 |
| RP5-908M14.9 | 0.46 (0.31–0.67) | 5.15E-05 |
| PART1 | 0.35 (0.21–0.59) | 5.71E-05 |
| DHRS4-AS1 | 0.48 (0.34–0.69) | 5.74E-05 |
| DANCR | 0.38 (0.24–0.61) | 5.89E-05 |
| AC068499.10 | 0.39 (0.25–0.63) | 7.81E-05 |
| AC097461.4 | 0.30 (0.16–0.54) | 7.83E-05 |
| RP11-266L9.8 | 0.41 (0.26–0.64) | 8.09E-05 |
| CTD-2199O4.6 | 0.46 (0.31–0.68) | 8.73E-05 |
| RP11-701H24.4 | 1.97 (1.40–2.76) | 9.73E-05 |
| RP11-282O18.3 | 2.36 (1.53–3.66) | 0.00011 |
| CTC-260E6.4 | 0.50 (0.35–0.71) | 0.00011 |
| AC012146.7 | 0.50 (0.35 – 0.71) | 0.00011 |
| CTD-2256P15.2 | 0.45 (0.30–0.68) | 0.00011 |
| RP11-298I3.3 | 0.47 (0.32–0.69) | 0.00012 |
| RP11-513G11.4 | 0.51 (0.36–0.72) | 0.00014 |
| RP11-634H22.1 | 0.50 (0.35–0.72) | 0.00015 |
| TRIM52-AS1 | 0.39 (0.24–0.63) | 0.00015 |
| RP11-51J9.5 | 0.41 (0.26–0.65) | 0.00016 |
| RP11-166P13.3 | 0.46 (0.31–0.69) | 0.00017 |
| RP11-264B14.2 | 2.62 (1.58–4.33) | 0.00018 |
| RP13-476E20.1 | 0.51 (0.35–0.72) | 0.00018 |
| RP3-337O18.9 | 2.03 (1.39–2.95) | 0.00022 |
| TOPORS-AS1 | 0.41 (0.25–0.66) | 0.00024 |
| RP11-91I8.3 | 0.53 (0.37–0.74) | 0.00026 |
| HCG14 | 0.53 (0.38–0.75) | 0.00028 |
| RP11-886P16.10 | 0.51 (0.35–0.73) | 0.00028 |
| RP11-895M11.3 | 0.52 (0.37–0.74) | 0.0003 |
| RP11-574K11.24 | 0.43 (0.27–0.68) | 0.00033 |
| RP11-127B20.3 | 0.48 (0.32–0.72) | 0.00033 |
| LINC01556 | 0.53 (0.38–0.75) | 0.00035 |
| ZNF561-AS1 | 0.52 (0.37–0.75) | 0.00035 |
| RP1-28H20.3 | 0.51 (0.35–0.74) | 0.00036 |
| RP11-500C11.3 | 0.52 (0.37–0.75) | 0.00038 |
| RP3-337H4.8 | 0.50 (0.34–0.73) | 0.00041 |
| RP11-96D1.8 | 1.97 (1.35–2.87) | 0.00043 |
| LINC00167 | 0.48 (0.32–0.73) | 0.00048 |
| RP11-554A11.9 | 1.87 (1.32–2.66) | 0.00049 |
| RP5-1074L1.1 | 0.51 (0.35–0.75) | 0.0005 |
| AC093702.1 | 0.48 (0.32–0.73) | 0.00052 |
| RP13-1032I1.7 | 0.52 (0.36–0.75) | 0.00053 |
| RP13-188A5.1 | 0.53 (0.37–0.76) | 0.00054 |
| RP11-573N10.1 | 2.30 (1.43–3.68) | 0.00054 |
| RP11-181C3.1 | 0.43 (0.27–0.70) | 0.00057 |
| RP1-232L24.3 | 0.53 (0.37–0.76) | 0.00062 |
| RP11-388C12.1 | 0.55 (0.39–0.78) | 0.00064 |
| PTOV1-AS2 | 2.23 (1.40–3.54) | 0.00068 |
| RP5-1065J22.8 | 0.47 (0.31–0.73) | 0.00071 |
| RP11-178L8.7 | 0.54 (0.38–0.77) | 0.00072 |
| RP11-69H7.2 | 1.80 (1.28–2.52) | 0.00072 |
| LINC00467 | 0.55 (0.39–0.78) | 0.00072 |
| CTB-36H16.2 | 2.27 (1.41–3.65) | 0.00077 |
| MIR762HG | 0.54 (0.38–0.77) | 0.00078 |
| RP11-629B11.5 | 0.47 (0.31–0.73) | 0.0008 |
| RP11-307C12.12 | 0.51 (0.34–0.76) | 0.00082 |
| RP3-337H4.10 | 0.48 (0.31–0.74) | 0.00083 |
| CTD-2553L13.10 | 0.48 (0.31–0.74) | 0.00084 |
| RP5-887A10.1 | 0.39 (0.23–0.68) | 0.00084 |
| RP11-421M1.8 | 0.47 (0.30–0.74) | 0.00093 |
| ZFHX4-AS1 | 2.12 (1.36–3.31) | 0.00093 |
| LINC00657 | 2.29 (1.40–3.75) | 0.00095 |
| RP11-66N11.8 | 0.56 (0.40–0.79) | 0.00099 |
| RP11-262H14.4 | 0.56 (0.40–0.79) | 0.00099 |
| MIR600HG | 1.79 (1.27–2.54) | 0.00101 |
| CTD-2619J13.5 | 2.19 (1.37–3.50) | 0.00103 |
| RP11-317P15.4 | 0.48 (0.31–0.74) | 0.00103 |
| C9orf41-AS1 | 0.49 (0.32–0.75) | 0.00104 |
| GS1-124K5.4 | 0.54 (0.37–0.78) | 0.00105 |
| AC007405.6 | 0.56 (0.39–0.79) | 0.00107 |
| LINC01524 | 1.78 (1.26–2.51) | 0.00108 |
| HM13-IT1 | 2.04 (1.33–3.12) | 0.00109 |
| LINC00664 | 0.55 (0.39–0.79) | 0.00117 |
| RP11-126K1.6 | 0.56 (0.39–0.79) | 0.00122 |
| CTB-113I20.2 | 0.55 (0.39–0.79) | 0.00125 |
| CTD-3128G10.6 | 2.12 (1.34–3.35) | 0.00126 |
| RP4-616B8.4 | 0.53 (0.36–0.78) | 0.00127 |
| RP11-314A20.2 | 0.57 (0.40–0.80) | 0.00131 |
| RP11-528I4.2 | 0.57 (0.41–0.81) | 0.00134 |
| RP11-196G11.5 | 0.57 (0.40–0.80) | 0.00134 |
| CACTIN-AS1 | 1.74 (1.24–2.44) | 0.00139 |
| RP5-937E21.8 | 0.57 (0.40–0.81) | 0.00147 |
| RP11-438L19.1 | 0.53 (0.36–0.79) | 0.00149 |
| RP4-695O20.1 | 1.87 (1.27–2.76) | 0.00157 |
| FARSA-AS1 | 1.74 (1.23–2.46) | 0.00159 |
| AC009005.2 | 0.57 (0.40–0.81) | 0.00163 |
| URB1-AS1 | 0.50 (0.33–0.77) | 0.00164 |
| RP11-686D22.4 | 0.56 (0.39–0.81) | 0.00166 |
| IDH1-AS1 | 0.57 (0.40–0.81) | 0.00167 |
| CTD-2256P15.3 | 0.55 (0.38–0.80) | 0.00167 |
| FOXP4-AS1 | 0.57 (0.40–0.81) | 0.0017 |
| AC018766.4 | 1.77 (1.24–2.52) | 0.0017 |
| RP11-385F7.1 | 0.58 (0.41–0.81) | 0.0017 |
| CTD-2132N18.2 | 1.79 (1.24–2.58) | 0.00178 |
| CTB-178M22.2 | 0.42 (0.24–0.72) | 0.00178 |
| AC079305.10 | 0.58 (0.41–0.82) | 0.00179 |
| SNHG19 | 0.52 (0.35–0.79) | 0.00183 |
| CTD-2537I9.13 | 2.11 (1.32–3.38) | 0.00184 |
| CTD-3065J16.9 | 0.58 (0.41–0.82) | 0.00186 |
| RP11-418H16.1 | 0.55 (0.38–0.80) | 0.00188 |
| CTA-384D8.34 | 0.54 (0.37–0.80) | 0.0019 |
| RP11-45P15.4 | 0.43 (0.25–0.73) | 0.00192 |
| ACAP2-IT1 | 1.87 (1.26–2.78) | 0.00193 |
| CTC-205M6.1 | 0.58 (0.41–0.82) | 0.00193 |
| SNHG9 | 0.51 (0.33–0.78) | 0.00195 |
| TYMSOS | 0.32 (0.16–0.66) | 0.002 |
| RP4-753F5.1 | 0.52 (0.35–0.79) | 0.00202 |
| LINC01483 | 0.58 (0.41–0.82) | 0.00204 |
| RP11-83A24.2 | 0.58 (0.41–0.82) | 0.00209 |
| RP11-33I11.2 | 0.47 (0.29–0.76) | 0.0021 |
| AC100830.5 | 1.99 (1.28–3.08) | 0.00213 |
| RP11-365H22.2 | 0.48 (0.30–0.77) | 0.00218 |
| RP11-785H5.1 | 0.57 (0.40–0.82) | 0.0022 |
| CTC-492K19.7 | 0.56 (0.39–0.81) | 0.00228 |
| RP3-402G11.27 | 1.87 (1.25–2.80) | 0.00229 |
| CTC-325H20.4 | 0.50 (0.32–0.78) | 0.00232 |
| CTD-2260A17.1 | 1.79 (1.23–2.61) | 0.00234 |
| UBXN10-AS1 | 0.57 (0.39–0.82) | 0.00236 |
| XXbac-B135H6.18 | 0.54 (0.36–0.80) | 0.00238 |
| RP11-81H14.2 | 0.59 (0.42–0.83) | 0.00238 |
| AC072062.3 | 0.57 (0.40–0.82) | 0.00239 |
| CTD-2162K18.4 | 0.59 (0.41–0.83) | 0.00239 |
| RP11-395A13.2 | 0.55 (0.37–0.81) | 0.0024 |
| LINC00261 | 0.56 (0.39–0.82) | 0.00242 |
| RP11-394I13.1 | 1.71 (1.21–2.42) | 0.00243 |
| RP11-225B17.2 | 0.57 (0.40–0.82) | 0.0025 |
| RP11-380B4.3 | 1.69 (1.20–2.37) | 0.0025 |
| DDX26B-AS1 | 0.55 (0.37–0.81) | 0.00256 |
| RP4-724E16.2 | 1.82 (1.23–2.68) | 0.00261 |
| XXbac-BPG252P9.10 | 0.43 (0.25–0.75) | 0.00263 |
| RP1-30M3.5 | 0.58 (0.40–0.83) | 0.00272 |
| RP11-383G6.3 | 2.01 (1.27–3.18) | 0.00278 |
| RP11-63A1.1 | 0.59 (0.41–0.83) | 0.00288 |
| AC015849.13 | 0.56 (0.39–0.82) | 0.00289 |
| RP5-963E22.6 | 0.56 (0.38–0.82) | 0.00293 |
| CTA-414D7.1 | 1.88 (1.24–2.85) | 0.00293 |
| B4GALT1-AS1 | 0.60 (0.43–0.84) | 0.00293 |
| AC007009.1 | 0.57 (0.39–0.83) | 0.00295 |
| RP11-603J24.5 | 1.88 (1.24–2.86) | 0.00296 |
| LINC00843 | 1.82 (1.23–2.70) | 0.00298 |
| CTD-3074O7.5 | 0.55 (0.37–0.82) | 0.00302 |
| RP11-557C18.4 | 0.59 (0.41–0.84) | 0.00303 |
| KB-1460A1.5 | 1.81 (1.22–2.67) | 0.00308 |
| CTD-2036P10.6 | 1.72 (1.20–2.47) | 0.00311 |
| RP1-111B22.3 | 1.72 (1.20–2.46) | 0.00312 |
| AC053503.4 | 0.56 (0.38–0.82) | 0.00319 |
| AC096559.1 | 0.59 (0.42–0.84) | 0.00327 |
| RP11-274B21.14 | 2.08 (1.28–3.40) | 0.00329 |
| RP11-359P5.1 | 1.84 (1.22–2.76) | 0.0033 |
| RP11-670E13.6 | 0.59 (0.42–0.84) | 0.00332 |
| CTD-2026K11.6 | 1.66 (1.18–2.34) | 0.00334 |
| XXbac-B461K10.4 | 1.87 (1.23–2.85) | 0.00336 |
| RP11-758H9.2 | 0.57 (0.39–0.83) | 0.00338 |
| LINC01006 | 0.57 (0.40–0.83) | 0.00338 |
| RP11-686D22.5 | 0.58 (0.40–0.83) | 0.00347 |
| RP4-816N1.6 | 0.60 (0.42–0.84) | 0.0035 |
| AC093673.5 | 0.59 (0.42–0.84) | 0.00351 |
| AC005154.6 | 0.54 (0.36–0.82) | 0.00352 |
| RP11-48B3.4 | 0.60 (0.43–0.85) | 0.00353 |
| RP11-1391J7.1 | 1.73 (1.20–2.50) | 0.00355 |
| RP11-415D17.3 | 0.56 (0.38–0.83) | 0.00359 |
| RP11-272P10.2 | 0.48 (0.30–0.79) | 0.00369 |
| RP11-490B18.5 | 0.59 (0.42–0.84) | 0.00373 |
| AP001439.2 | 0.59 (0.42–0.84) | 0.00376 |
| RP11-677I18.3 | 0.55 (0.37–0.83) | 0.00376 |
| CTC-338M12.5 | 0.59 (0.42–0.85) | 0.00381 |
| RP11-662B19.2 | 0.59 (0.42–0.85) | 0.00382 |
| RP11-390E23.6 | 0.60 (0.43–0.85) | 0.00383 |
| NNT-AS1 | 0.58 (0.40–0.84) | 0.00384 |
| RP13-225O21.2 | 1.83 (1.21–2.75) | 0.00386 |
| RP11-432I5.2 | 0.50 (0.31–0.80) | 0.00388 |
| RP11-352D13.6 | 2.02 (1.25–3.26) | 0.00388 |
| FOCAD-AS1 | 0.49 (0.30–0.79) | 0.00389 |
| RP11-632L2.2 | 1.78 (1.20–2.64) | 0.00395 |
| CTD-2162K18.3 | 0.61 (0.43–0.85) | 0.00395 |
| RP11-307C12.11 | 0.60 (0.43–0.85) | 0.00396 |
| RP1-40E16.12 | 0.60 (0.43–0.85) | 0.00405 |
| LINC01094 | 1.94 (1.23–3.05) | 0.00416 |
| RP11-110G21.1 | 0.61 (0.43–0.85) | 0.00416 |
| RP11-675F6.3 | 0.58 (0.40–0.84) | 0.00423 |
| RP11-475A13.1 | 1.94 (1.23–3.07) | 0.00424 |
| RP5-884G6.2 | 0.60 (0.42–0.85) | 0.00431 |
| RP11-229P13.25 | 0.53 (0.34–0.82) | 0.00434 |
| RP11-77H9.8 | 0.59 (0.41–0.85) | 0.00435 |
| HAGLROS | 0.53 (0.35–0.82) | 0.00438 |
| UBA6-AS1 | 0.46 (0.27–0.78) | 0.00441 |
| RP11-517B11.4 | 0.60 (0.42–0.85) | 0.00444 |
| PITRM1-AS1 | 1.67 (1.17–2.38) | 0.00447 |
| RP1-234P15.4 | 0.59 (0.41–0.85) | 0.00449 |
| GAS5 | 1.66 (1.17–2.35) | 0.00449 |
| PCBP2-OT1 | 1.77 (1.19–2.63) | 0.0045 |
| CTD-2537I9.12 | 1.99 (1.24–3.21) | 0.00452 |
| AC090587.4 | 2.09 (1.26–3.47) | 0.00452 |
| RP11-65N13.8 | 0.60 (0.42–0.85) | 0.00453 |
| RP11-108L7.15 | 0.60 (0.42–0.85) | 0.00464 |
| CTD-2256P15.1 | 0.45 (0.26–0.78) | 0.00466 |
| CTB-133G6.2 | 0.61 (0.43–0.86) | 0.00468 |
| AC005578.3 | 0.61 (0.43–0.86) | 0.0047 |
| CTD-2325M2.1 | 0.55 (0.36–0.83) | 0.00472 |
| RP4-614O4.11 | 1.81 (1.20–2.73) | 0.00473 |
| RP11-467D18.2 | 2.06 (1.25–3.40) | 0.00475 |
| PAXIP1-AS1 | 0.51 (0.31–0.81) | 0.00475 |
| YTHDF3-AS1 | 0.48 (0.29–0.80) | 0.00483 |
| RP11-158I9.8 | 0.49 (0.30–0.81) | 0.00486 |
| RP11-483F11.7 | 0.39 (0.20–0.75) | 0.00487 |
| RP11-464F9.21 | 1.62 (1.16–2.28) | 0.00491 |
| RP11-393I2.4 | 0.55 (0.36–0.83) | 0.00491 |
| RP11-481J2.3 | 0.58 (0.39–0.85) | 0.00492 |
| RP11-160O5.1 | 0.49 (0.30–0.81) | 0.00498 |
| RP11-599B13.7 | 0.58 (0.40–0.85) | 0.00498 |
| RP11-234K24.6 | 1.65 (1.16–2.34) | 0.00504 |
| LINC01550 | 0.59 (0.41–0.85) | 0.00507 |
| RP11-690I21.2 | 1.62 (1.16–2.28) | 0.0051 |
| CTC-209H22.3 | 0.47 (0.28–0.80) | 0.00516 |
| RP11-203J24.8 | 1.63 (1.16–2.28) | 0.00516 |
| CTD-2540B15.6 | 1.87 (1.21–2.89) | 0.00516 |
| AP001462.6 | 0.54 (0.35–0.83) | 0.00527 |
| RP11-505K9.1 | 0.61 (0.43–0.86) | 0.0053 |
| ZNF205-AS1 | 0.40 (0.21–0.76) | 0.00531 |
| GS1-124K5.11 | 1.73 (1.18–2.55) | 0.00535 |
| AP000704.5 | 0.48 (0.29–0.81) | 0.00537 |
| RP11-48G14.3 | 0.50 (0.31–0.82) | 0.0054 |
| RP11-717H13.1 | 0.45 (0.26–0.79) | 0.00543 |
| RP6-65G23.3 | 0.62 (0.44–0.87) | 0.00554 |
| CTC-510F12.3 | 0.62 (0.44–0.87) | 0.00555 |
| RP13-516M14.8 | 1.62 (1.15–2.28) | 0.00559 |
| RP11-227G15.12 | 0.52 (0.32–0.82) | 0.00563 |
| CTC-444N24.8 | 0.56 (0.37–0.84) | 0.00563 |
| RP11-301O19.1 | 0.43 (0.24–0.78) | 0.00567 |
| RP11-226L15.5 | 0.59 (0.41–0.86) | 0.00574 |
| AC009403.2 | 0.57 (0.38–0.85) | 0.00574 |
| RP11-402D21.2 | 1.99 (1.22–3.24) | 0.00578 |
| RP11-10A14.4 | 0.61 (0.44–0.87) | 0.00579 |
| RP11-253E3.3 | 1.72 (1.17–2.53) | 0.00582 |
| RP11-1000B6.5 | 0.46 (0.26–0.80) | 0.00586 |
| RP11-258C19.7 | 2.06 (1.23–3.44) | 0.00586 |
| RP11-110A12.2 | 0.56 (0.38–0.85) | 0.00594 |
| RP5-1136G13.2 | 0.42 (0.23–0.78) | 0.00594 |
| RP11-120E11.2 | 0.61 (0.43–0.87) | 0.00598 |
| RP11-111J6.2 | 0.62 (0.44–0.87) | 0.00601 |
| RP11-359D14.2 | 1.71 (1.17–2.50) | 0.00601 |
| PWAR5 | 1.69 (1.16–2.45) | 0.00601 |
| AP000569.9 | 0.60 (0.42–0.87) | 0.00606 |
| ARHGAP26-AS1 | 0.52 (0.32–0.83) | 0.00609 |
| AC000123.2 | 1.66 (1.15–2.38) | 0.0061 |
| CCDC147-AS1 | 0.60 (0.41–0.86) | 0.0061 |
| RP11-758P17.3 | 0.51 (0.31–0.82) | 0.0061 |
| RP11-632F7.3 | 1.78 (1.18–2.69) | 0.00618 |
| RP1-251I12.1 | 0.41 (0.21–0.77) | 0.0062 |
| CTC-260E6.3 | 0.62 (0.44–0.87) | 0.00632 |
| CACNA1C-AS1 | 1.76 (1.17–2.65) | 0.00645 |
| LNX1-AS2 | 0.41 (0.21–0.78) | 0.00651 |
| AC104667.3 | 0.62 (0.44–0.88) | 0.00658 |
| CTD-2310F14.1 | 0.62 (0.44–0.87) | 0.00664 |
| RP11-752G15.10 | 1.60 (1.14–2.25) | 0.00664 |
| RP11-314N13.9 | 1.72 (1.16–2.54) | 0.00667 |
| RP11-706O15.1 | 0.59 (0.40–0.86) | 0.00667 |
| RP4-761J14.9 | 0.60 (0.42–0.87) | 0.00675 |
| ZSCAN16-AS1 | 0.62 (0.44–0.88) | 0.00682 |
| CTC-444N24.7 | 0.62 (0.44–0.88) | 0.00684 |
| SCOC-AS1 | 0.61 (0.43–0.87) | 0.00686 |
| RP11-539L10.3 | 0.63 (0.44–0.88) | 0.00689 |
| RP11-872J21.3 | 0.42 (0.23–0.79) | 0.00696 |
| RP11-214K3.21 | 2.02 (1.21–3.36) | 0.00701 |
| ROR1-AS1 | 0.52 (0.33–0.84) | 0.0072 |
| RP11-552M6.1 | 1.86 (1.18–2.93) | 0.0073 |
| RP11-337N6.3 | 2.53 (1.28–4.99) | 0.00735 |
| RP5-1116H23.3 | 2.01 (1.20–3.34) | 0.00746 |
| RP11-881M11.1 | 0.44 (0.24–0.80) | 0.00747 |
| RP11-562A8.4 | 1.71 (1.15–2.54) | 0.00752 |
| RP11-51J9.6 | 0.62 (0.43–0.88) | 0.00757 |
| CTD-3193O13.12 | 0.54 (0.34–0.85) | 0.00761 |
| RP11-386I14.4 | 0.60 (0.41–0.87) | 0.00776 |
| RP13-582O9.5 | 0.59 (0.40–0.87) | 0.00784 |
| RP1-315G1.3 | 0.60 (0.41–0.87) | 0.00785 |
| RP11-778D9.13 | 0.63 (0.45–0.89) | 0.00787 |
| AP001062.9 | 0.40 (0.20–0.78) | 0.00787 |
| RP5-855D21.2 | 1.95 (1.19–3.20) | 0.00793 |
| RP1-178F10.1 | 1.67 (1.14–2.45) | 0.00795 |
| RP11-469H8.6 | 0.53 (0.33–0.85) | 0.00796 |
| CTD-2302E22.6 | 0.62 (0.44–0.88) | 0.008 |
| RP11-73E17.2 | 0.63 (0.45–0.89) | 0.008 |
| RP11-540B6.6 | 1.67 (1.14–2.44) | 0.00802 |
| STX17-AS1 | 0.52 (0.32–0.84) | 0.00803 |
| AC091177.1 | 1.64 (1.14–2.36) | 0.00804 |
| CTD-2007H13.3 | 0.58 (0.39–0.87) | 0.00805 |
| RP4-612B15.3 | 0.43 (0.23–0.80) | 0.00809 |
| ATP2A1-AS1 | 0.55 (0.36–0.86) | 0.00809 |
| RP11-422P24.10 | 0.63 (0.45–0.89) | 0.00813 |
| RP11-434H6.7 | 1.84 (1.17–2.89) | 0.00813 |
| AC079807.4 | 0.63 (0.45–0.89) | 0.00822 |
| RP11-799B12.1 | 0.63 (0.44–0.89) | 0.00826 |
| RP11-1114A5.4 | 0.61 (0.42–0.88) | 0.00833 |
| RP11-20E24.1 | 0.59 (0.40–0.87) | 0.00833 |
| RP11-579D7.4 | 0.55 (0.36–0.86) | 0.00834 |
| RP11-672L10.6 | 0.63 (0.45–0.89) | 0.00837 |
| FLNB-AS1 | 1.62 (1.13–2.31) | 0.00841 |
| RP11-483L5.1 | 0.53 (0.33–0.85) | 0.00849 |
| DNAJC27-AS1 | 0.47 (0.27–0.83) | 0.00856 |
| CDC37L1-AS1 | 0.62 (0.44–0.89) | 0.0086 |
| FLJ37453 | 0.60 (0.41–0.88) | 0.00861 |
| AC009961.3 | 0.63 (0.44–0.89) | 0.00864 |
| HCG16 | 0.61 (0.43–0.88) | 0.00865 |
| RP11-25G10.2 | 0.47 (0.27–0.83) | 0.00867 |
| NEAT1 | 1.94 (1.18–3.18) | 0.00872 |
| RP11-1152H14.1 | 0.62 (0.43–0.89) | 0.00873 |
| LINC01456 | 0.64 (0.45–0.89) | 0.0088 |
| JARID2-AS1 | 0.63 (0.45–0.89) | 0.00886 |
| PPP4R1-AS1 | 0.63 (0.44–0.89) | 0.0089 |
| RP11-73K9.2 | 0.62 (0.44–0.89) | 0.00892 |
| CH507-338C24.3 | 0.53 (0.33–0.85) | 0.00897 |
| CTD-2287O16.5 | 0.63 (0.45–0.89) | 0.00902 |
| PRR34-AS1 | 1.59 (1.12–2.26) | 0.00904 |
| RP11-676J12.9 | 1.63 (1.13–2.34) | 0.00907 |
| AC004076.5 | 0.57 (0.37–0.87) | 0.00908 |
| RP4-671O14.6 | 2.21 (1.22–4.01) | 0.00913 |
| RP11-944L7.4 | 2.09 (1.20–3.65) | 0.00913 |
| RP11-126K1.2 | 0.64 (0.46–0.89) | 0.0092 |
| RP11-148B6.1 | 0.60 (0.41–0.88) | 0.00925 |
| RP13-650J16.1 | 0.61 (0.42–0.88) | 0.00934 |
| RP11-240G22.5 | 1.59 (1.12–2.26) | 0.00939 |
| bP-21201H5.2 | 0.51 (0.31–0.85) | 0.00943 |
| RP11-504G3.2 | 1.81 (1.16–2.82) | 0.00948 |
| RP11-1094M14.11 | 0.64 (0.46–0.90) | 0.00949 |
| AC100830.3 | 1.56 (1.11–2.19) | 0.00953 |
| RP11-261C10.5 | 0.63 (0.45–0.90) | 0.00955 |
| RP11-110I1.6 | 0.57 (0.37–0.87) | 0.00974 |
| RP11-316M21.7 | 0.62 (0.43–0.89) | 0.00975 |
| LCMT1-AS1 | 0.61 (0.42–0.89) | 0.00977 |
| RP11-122K13.12 | 2.26 (1.22–4.21) | 0.00992 |
| AC010894.3 | 0.47 (0.26–0.84) | 0.01026 |
| AC068831.10 | 0.58 (0.38–0.88) | 0.01033 |
| RP1-267L14.6 | 1.57 (1.11–2.21) | 0.01037 |
| RP11-835E18.5 | 1.95 (1.17–3.26) | 0.01047 |
| RP11-227G15.2 | 1.83 (1.15–2.91) | 0.01049 |
| MYCNOS | 0.54 (0.33–0.86) | 0.01062 |
| RP5-1132H15.1 | 1.62 (1.12–2.35) | 0.01068 |
| RP11-849H4.4 | 1.85 (1.15–2.97) | 0.0107 |
| BACE1-AS | 0.52 (0.31–0.86) | 0.01081 |
| CTD-2095E4.3 | 1.82 (1.15–2.89) | 0.01081 |
| RP11-701H24.3 | 2.16 (1.19–3.91) | 0.01082 |
| CTC-463A16.1 | 0.47 (0.26–0.84) | 0.01088 |
| AC009506.1 | 0.64 (0.45–0.90) | 0.01098 |
| RP1-100J12.1 | 0.54 (0.33–0.87) | 0.01102 |
| RP11-214K3.23 | 1.98 (1.17–3.36) | 0.01103 |
| RP11-421E14.2 | 0.59 (0.39–0.89) | 0.01108 |
| RP11-214K3.22 | 1.59 (1.11–2.28) | 0.01109 |
| RP11-761E20.1 | 1.56 (1.11–2.19) | 0.01115 |
| RP11-1072A3.4 | 0.63 (0.44–0.90) | 0.01117 |
| RP3-508I15.19 | 1.58 (1.11–2.25) | 0.01121 |
| AC005757.6 | 1.59 (1.11–2.27) | 0.01126 |
| RP11-91G21.1 | 0.58 (0.38–0.88) | 0.01127 |
| CAMTA1-IT1 | 0.50 (0.29–0.85) | 0.01128 |
| RP11-961A15.3 | 0.57 (0.37–0.88) | 0.01132 |
| ZNF503-AS2 | 0.45 (0.24–0.83) | 0.0114 |
| RP11-732A21.3 | 0.51 (0.30–0.86) | 0.01151 |
| RP11-178H8.7 | 1.86 (1.15–3.00) | 0.01152 |
| RBAKDN | 1.55 (1.10–2.18) | 0.01159 |
| RP5-1050D4.4 | 1.55 (1.10–2.18) | 0.0116 |
| AC013460.1 | 0.63 (0.45–0.90) | 0.01166 |
| RP11-524O1.4 | 0.62 (0.43–0.90) | 0.01169 |
| RP11-872J21.5 | 0.55 (0.34–0.88) | 0.01177 |
| RP11-527L4.2 | 0.56 (0.36–0.88) | 0.01178 |
| AC068282.3 | 0.61 (0.42–0.90) | 0.01181 |
| LL0XNC01-237H1.3 | 1.56 (1.10–2.20) | 0.01181 |
| RP11-121C2.2 | 0.61 (0.42–0.90) | 0.01187 |
| RP11-12G12.7 | 0.64 (0.46–0.91) | 0.01191 |
| KB-1674E1.2 | 0.55 (0.35–0.88) | 0.01193 |
| RP11-97O12.6 | 1.83 (1.14–2.92) | 0.01194 |
| RP11-21B21.4 | 1.61 (1.11–2.35) | 0.01202 |
| KB-1208A12.3 | 0.65 (0.46–0.91) | 0.01205 |
| CTD-2609K8.3 | 1.55 (1.10–2.18) | 0.0121 |
| RP4-545L17.11 | 0.61 (0.41–0.90) | 0.01211 |
| RP11-278C7.5 | 1.78 (1.13–2.79) | 0.01212 |
| BBOX1-AS1 | 0.65 (0.46–0.91) | 0.01214 |
| SERTAD4-AS1 | 0.62 (0.42–0.90) | 0.01217 |
| RP11-6B19.2 | 1.75 (1.13–2.70) | 0.01228 |
| PRMT5-AS1 | 1.96 (1.16–3.31) | 0.01231 |
| RP11-141B14.2 | 1.54 (1.10–2.15) | 0.01237 |
| RP1-313I6.12 | 0.44 (0.23–0.84) | 0.01243 |
| RNASEH1-AS1 | 0.54 (0.33–0.88) | 0.01248 |
| LINC00869 | 0.65 (0.46–0.91) | 0.0125 |
| CTC-203F4.2 | 0.65 (0.46–0.91) | 0.01252 |
| CH17-118O6.2 | 0.65 (0.46–0.91) | 0.01257 |
| RP11-686D22.8 | 0.63 (0.44–0.91) | 0.01272 |
| KB-1043D8.6 | 0.60 (0.40–0.90) | 0.01273 |
| CTC-471J1.11 | 0.64 (0.45–0.91) | 0.0128 |
| RP11-84A14.4 | 0.61 (0.41–0.90) | 0.01298 |
| RP11-332M2.1 | 0.53 (0.32–0.87) | 0.01299 |
| CTD-3193K9.3 | 1.60 (1.10–2.33) | 0.01303 |
| RP11-319G6.3 | 1.53 (1.09–2.15) | 0.01305 |
| CTD-2020K17.4 | 0.65 (0.46–0.91) | 0.01313 |
| AC034243.1 | 0.61 (0.41–0.90) | 0.01313 |
| EPB41L4A-AS1 | 1.54 (1.09–2.16) | 0.01329 |
| CTD-2622I13.3 | 1.74 (1.12–2.70) | 0.01332 |
| RP4-604K5.2 | 0.52 (0.31–0.87) | 0.01332 |
| DDX39B-AS1 | 0.62 (0.43–0.91) | 0.01333 |
| ADAMTS9-AS1 | 2.35 (1.19–4.63) | 0.0134 |
| CTA-14H9.5 | 0.62 (0.42–0.91) | 0.0134 |
| ZFAS1 | 1.53 (1.09–2.15) | 0.01341 |
| RP11-247A12.2 | 0.58 (0.38–0.89) | 0.01345 |
| RP11-617F23.2 | 0.55 (0.34–0.88) | 0.01345 |
| KB-431C1.5 | 1.64 (1.11–2.44) | 0.01349 |
| RP11-323J4.1 | 0.64 (0.45–0.91) | 0.01361 |
| LINC01420 | 0.59 (0.39–0.90) | 0.01364 |
| RAD51-AS1 | 1.66 (1.11–2.48) | 0.01387 |
| RP11-661C3.2 | 0.59 (0.39–0.90) | 0.0139 |
| LL0XNC01-36H8.1 | 1.53 (1.09–2.14) | 0.01399 |
| RP11-436D23.1 | 1.82 (1.13–2.94) | 0.014 |
| AP003068.9 | 0.64 (0.45–0.92) | 0.01415 |
| CTD-2373H9.5 | 1.53 (1.09–2.15) | 0.01422 |
| RP11-61L19.3 | 0.56 (0.35–0.89) | 0.01431 |
| RP11-278J6.4 | 0.47 (0.26–0.86) | 0.0144 |
| TIPARP-AS1 | 0.60 (0.40–0.90) | 0.01442 |
| RP11-44M6.7 | 1.53 (1.09–2.16) | 0.01442 |
| RP11-973H7.3 | 0.63 (0.44–0.91) | 0.01444 |
| RP11-74D7.3 | 0.65 (0.47–0.92) | 0.0145 |
| RP3-402G11.28 | 1.92 (1.14–3.23) | 0.01457 |
| AP001469.5 | 1.64 (1.10–2.45) | 0.01461 |
| RP11-323F24.3 | 0.59 (0.39–0.90) | 0.01465 |
| DEPDC1-AS1 | 0.57 (0.36–0.90) | 0.01469 |
| RP11-101E13.5 | 0.65 (0.47–0.92) | 0.01471 |
| LA16c-316G12.2 | 1.75 (1.12–2.75) | 0.0148 |
| LINC00240 | 0.62 (0.42–0.91) | 0.01482 |
| DLG5-AS1 | 0.63 (0.44–0.91) | 0.01482 |
| RP13-766D20.2 | 1.53 (1.09–2.15) | 0.01485 |
| RP11-1275H24.3 | 0.64 (0.45–0.92) | 0.01489 |
| CTB-193M12.5 | 0.61 (0.40–0.91) | 0.01496 |
| RP11-792A8.4 | 0.65 (0.46–0.92) | 0.01499 |
| RP11-722E23.2 | 0.58 (0.37–0.90) | 0.01509 |
| RP11-121M22.1 | 0.53 (0.32–0.88) | 0.0151 |
| RP11-502I4.3 | 0.66 (0.47–0.92) | 0.01513 |
| USP2-AS1 | 0.45 (0.24–0.86) | 0.01533 |
| RP11-268G13.1 | 0.65 (0.46–0.92) | 0.01544 |
| MAST4-AS1 | 0.47 (0.25–0.86) | 0.01549 |
| AC034220.3 | 0.62 (0.42–0.91) | 0.0155 |
| LINC01183 | 0.65 (0.46–0.92) | 0.01552 |
| RP11-756P10.6 | 0.61 (0.41–0.91) | 0.01569 |
| LINCMD1 | 0.45 (0.24–0.86) | 0.01574 |
| SEPSECS-AS1 | 0.47 (0.25–0.87) | 0.01583 |
| CECR5-AS1 | 0.66 (0.47–0.92) | 0.01584 |
| RP11-10N23.2 | 1.67 (1.10–2.54) | 0.01585 |
| HOXD-AS2 | 0.54 (0.33–0.89) | 0.01592 |
| RP11-394B2.1 | 1.58 (1.09–2.29) | 0.01594 |
| RP11-63L7.5 | 0.62 (0.43–0.92) | 0.016 |
| AC026904.1 | 1.51 (1.08–2.12) | 0.01604 |
| RP11-6O2.3 | 1.68 (1.10–2.55) | 0.01608 |
| RP11-96C23.9 | 1.82 (1.12–2.97) | 0.01615 |
| RP4-671G15.2 | 0.66 (0.47–0.93) | 0.01622 |
| RP11-667K14.5 | 1.72 (1.11–2.68) | 0.01622 |
| JPX | 0.60 (0.40–0.91) | 0.01631 |
| CTA-363E19.2 | 1.52 (1.08–2.13) | 0.01637 |
| RP11-752G15.8 | 1.59 (1.09–2.32) | 0.01651 |
| RP11-25C19.3 | 0.51 (0.29–0.88) | 0.01652 |
| AC000123.4 | 1.58 (1.09–2.30) | 0.01654 |
| RP11-303E16.2 | 0.65 (0.46–0.93) | 0.01656 |
| PDCD4-AS1 | 0.63 (0.44–0.92) | 0.01663 |
| RP11-334G22.1 | 0.66 (0.47–0.93) | 0.01671 |
| EHMT2-AS1 | 1.84 (1.12–3.03) | 0.01677 |
| RP11-356J5.12 | 0.49 (0.28–0.88) | 0.01677 |
| RP11-80H5.2 | 0.57 (0.36–0.90) | 0.01688 |
| AC011290.4 | 0.66 (0.47–0.93) | 0.01691 |
| RP11-7K24.3 | 1.65 (1.09–2.49) | 0.01695 |
| RP11-1099M24.6 | 1.55 (1.08–2.23) | 0.017 |
| RP11-295G20.2 | 0.61 (0.40–0.91) | 0.01712 |
| RP11-676J12.8 | 0.66 (0.47–0.93) | 0.01712 |
| SAP30L-AS1 | 0.63 (0.43–0.92) | 0.01715 |
| HCG18 | 0.66 (0.47–0.93) | 0.01716 |
| RP11-57A19.2 | 0.62 (0.41–0.92) | 0.01719 |
| RP11-481J2.4 | 0.62 (0.41–0.92) | 0.01722 |
| AC004540.4 | 0.61 (0.41–0.92) | 0.01726 |
| HS1BP3-IT1 | 1.51 (1.08–2.13) | 0.01744 |
| AC016700.5 | 0.59 (0.39–0.91) | 0.01746 |
| RP11-144I2.1 | 1.83 (1.11–3.00) | 0.01754 |
| RP11-203B9.4 | 0.57 (0.36–0.91) | 0.01758 |
| MYLK-AS1 | 0.60 (0.39–0.91) | 0.01762 |
| RP11-750H9.5 | 1.64 (1.09–2.47) | 0.01763 |
| RP11-53O19.1 | 0.62 (0.42–0.92) | 0.01766 |
| RP5-991G20.1 | 0.55 (0.33–0.90) | 0.01778 |
| STX18-AS1 | 0.65 (0.46–0.93) | 0.01782 |
| RP11-93H24.3 | 1.53 (1.08–2.17) | 0.01786 |
| C1orf147 | 1.74 (1.10–2.77) | 0.01796 |
| AC026471.6 | 0.65 (0.46–0.93) | 0.01797 |
| RP4-665J23.1 | 2.00 (1.13–3.54) | 0.01804 |
| FAM83H-AS1 | 0.66 (0.47–0.93) | 0.01812 |
| CTD-3126B10.2 | 1.74 (1.10–2.76) | 0.01828 |
| STEAP3-AS1 | 1.57 (1.08–2.28) | 0.01831 |
| RP11-35P15.1 | 0.65 (0.46–0.93) | 0.01844 |
| RP11-474D1.2 | 0.51 (0.29–0.89) | 0.0186 |
| RP4-773N10.4 | 1.52 (1.07–2.15) | 0.01862 |
| RP11-946P6.6 | 1.50 (1.07–2.10) | 0.01863 |
| RAD21-AS1 | 0.63 (0.43–0.93) | 0.01869 |
| RP11-566E18.1 | 1.58 (1.08–2.32) | 0.01871 |
| RP11-456D7.1 | 0.58 (0.37–0.91) | 0.01873 |
| RP5-858B6.3 | 0.54 (0.33–0.90) | 0.01873 |
| AL022341.3 | 0.67 (0.48–0.93) | 0.01876 |
| RP11-372B4.3 | 0.64 (0.44–0.93) | 0.01877 |
| RP11-546B15.1 | 1.51 (1.07–2.14) | 0.01882 |
| AC093110.3 | 1.51 (1.07–2.12) | 0.01884 |
| RP11-1109M24.5 | 1.50 (1.07–2.10) | 0.01892 |
| RP4-530I15.9 | 1.65 (1.09–2.50) | 0.019 |
| RP1-228P16.8 | 0.55 (0.33–0.91) | 0.01901 |
| RP11-498C9.12 | 1.67 (1.09–2.56) | 0.01914 |
| RP11-70L8.4 | 1.70 (1.09–2.66) | 0.01917 |
| TSTD3 | 0.66 (0.46–0.93) | 0.01921 |
| AC004041.2 | 1.54 (1.07–2.22) | 0.01928 |
| RP11-707P17.2 | 1.70 (1.09–2.64) | 0.01957 |
| RP11-1007I13.4 | 1.50 (1.07–2.10) | 0.01957 |
| GS1-279B7.1 | 0.64 (0.43–0.93) | 0.01958 |
| AC002310.12 | 0.61 (0.40–0.92) | 0.01959 |
| RP11-400F19.6 | 1.89 (1.11–3.24) | 0.01961 |
| CTC-339F2.2 | 1.58 (1.07–2.31) | 0.01996 |
| AC009120.6 | 1.53 (1.07–2.20) | 0.01999 |
| RP13-507P19.1 | 1.74 (1.09–2.78) | 0.02002 |
| RP1-8B1.4 | 0.67 (0.48–0.94) | 0.02013 |
| OSGEPL1-AS1 | 0.67 (0.47–0.94) | 0.02014 |
| CSNK1G2-AS1 | 1.49 (1.06–2.09) | 0.02032 |
| RP11-63M22.1 | 0.61 (0.40–0.93) | 0.0204 |
| AC007787.3 | 1.79 (1.09–2.92) | 0.02047 |
| RP11-881L2.1 | 0.61 (0.40–0.93) | 0.02051 |
| RP11-958F21.1 | 1.72 (1.09–2.73) | 0.0206 |
| C22orf24 | 0.51 (0.29–0.90) | 0.02062 |
| RP11-484D2.5 | 0.60 (0.39–0.93) | 0.02074 |
| CARS-AS1 | 1.64 (1.08–2.49) | 0.02076 |
| RP11-620J15.1 | 1.56 (1.07–2.29) | 0.02084 |
| RP11-864J10.4 | 1.53 (1.07–2.20) | 0.02091 |
| AC002451.3 | 0.54 (0.32–0.91) | 0.02097 |
| DKFZP434I0714 | 0.67 (0.47–0.94) | 0.02101 |
| RP3-370M22.8 | 0.65 (0.45–0.94) | 0.02102 |
| AC005523.3 | 0.54 (0.32–0.91) | 0.02111 |
| RP11-1167A19.2 | 0.64 (0.44–0.94) | 0.02113 |
| AF064858.10 | 0.63 (0.42–0.93) | 0.0212 |
| AC137934.1 | 0.56 (0.35–0.92) | 0.02124 |
| RP11-855A2.2 | 0.50 (0.27–0.90) | 0.02125 |
| RP11-126L15.4 | 1.73 (1.08–2.76) | 0.02131 |
| RP11-390K5.6 | 1.69 (1.08–2.63) | 0.02133 |
| RP11-692D12.1 | 0.64 (0.44–0.94) | 0.02147 |
| RP11-346C20.3 | 0.64 (0.44–0.94) | 0.02161 |
| RP11-252K23.2 | 0.65 (0.45–0.94) | 0.02163 |
| RP11-114F10.3 | 0.55 (0.33–0.92) | 0.02166 |
| RP11-715J22.6 | 1.53 (1.06–2.20) | 0.0217 |
| RP11-620J15.3 | 0.64 (0.43–0.94) | 0.02177 |
| CTB-50L17.9 | 1.58 (1.07–2.35) | 0.02178 |
| RP11-45M22.3 | 1.49 (1.06–2.10) | 0.02183 |
| CTD-2339F6.1 | 1.49 (1.06–2.10) | 0.02183 |
| RP11-563N4.1 | 0.57 (0.35–0.92) | 0.02186 |
| AL133243.2 | 0.45 (0.23–0.89) | 0.02193 |
| RP1-197B17.5 | 0.58 (0.37–0.93) | 0.02193 |
| RP11-575A19.2 | 1.79 (1.09–2.94) | 0.02193 |
| TOB1-AS1 | 0.65 (0.45–0.94) | 0.02195 |
| CTB-138E5.1 | 0.67 (0.47–0.94) | 0.02196 |
| RP11-150O12.6 | 0.50 (0.28–0.90) | 0.02203 |
| RP11-54G14.1 | 1.57 (1.07–2.32) | 0.02217 |
| CTD-2144E22.8 | 1.79 (1.09–2.96) | 0.02217 |
| LINC00265 | 1.54 (1.06–2.23) | 0.02218 |
| RP11-545G3.1 | 0.60 (0.39–0.93) | 0.02231 |
| RP11-157J24.2 | 1.58 (1.07–2.34) | 0.02231 |
| ST7-AS1 | 0.51 (0.29–0.91) | 0.02233 |
| RP11-98D18.9 | 0.63 (0.43–0.94) | 0.02261 |
| RP11-73M18.10 | 1.61 (1.07–2.42) | 0.02262 |
| NPTN-IT1 | 1.57 (1.07–2.32) | 0.02276 |
| RP11-261N11.8 | 1.63 (1.07–2.48) | 0.02283 |
| RP11-90M2.5 | 1.72 (1.08–2.75) | 0.02294 |
| RASSF1-AS1 | 0.55 (0.33–0.92) | 0.02298 |
| LINC01003 | 0.67 (0.48–0.95) | 0.02299 |
| AC133528.2 | 0.67 (0.48–0.95) | 0.02304 |
| LINC01184 | 0.67 (0.48–0.95) | 0.02309 |
| LINC00997 | 0.55 (0.33–0.92) | 0.02321 |
| RP11-372K14.2 | 0.65 (0.45–0.94) | 0.02324 |
| RP5-1142A6.9 | 1.54 (1.06–2.24) | 0.02326 |
| RP11-446N19.1 | 0.64 (0.44–0.94) | 0.02329 |
| RBM12B-AS1 | 1.64 (1.07–2.53) | 0.02338 |
| RP4-798A10.7 | 0.64 (0.44–0.94) | 0.0234 |
| RP11-278C7.3 | 1.53 (1.06–2.20) | 0.02352 |
| RP11-972P1.10 | 0.58 (0.36–0.93) | 0.02353 |
| RP11-20I23.2 | 1.87 (1.09–3.20) | 0.02353 |
| LINC00998 | 0.65 (0.45–0.94) | 0.02365 |
| CTD-2349P21.10 | 1.52 (1.06–2.18) | 0.02366 |
| RP11-839D17.3 | 0.59 (0.38–0.93) | 0.0237 |
| RP3-337H4.9 | 0.65 (0.45–0.94) | 0.02381 |
| RP11-95D17.1 | 0.65 (0.45–0.95) | 0.02383 |
| RP11-466A19.1 | 1.54 (1.06–2.23) | 0.02387 |
| RP11-155D18.13 | 0.58 (0.36–0.93) | 0.0239 |
| AC004623.3 | 1.48 (1.05–2.08) | 0.02399 |
| ANKRD33B-AS1 | 1.48 (1.05–2.07) | 0.02403 |
| RP11-139H14.5 | 1.66 (1.07–2.58) | 0.02404 |
| RP11-806H10.4 | 0.62 (0.41–0.94) | 0.02405 |
| RP1-302G2.5 | 0.62 (0.41–0.94) | 0.02411 |
| RP11-3P17.5 | 1.51 (1.06–2.16) | 0.02415 |
| RP13-942N8.1 | 0.57 (0.35–0.93) | 0.02424 |
| RP11-50C13.1 | 1.59 (1.06–2.37) | 0.02429 |
| RP11-575L7.8 | 1.56 (1.06–2.30) | 0.02439 |
| RP11-235E17.3 | 1.57 (1.06–2.32) | 0.0244 |
| RP11-524F11.2 | 0.68 (0.48–0.95) | 0.0244 |
| AC142119.1 | 0.64 (0.43–0.94) | 0.02441 |
| RP11-513I15.6 | 1.65 (1.07–2.54) | 0.02442 |
| SNHG8 | 1.48 (1.05–2.09) | 0.02443 |
| RP11-253A20.1 | 0.48 (0.25–0.91) | 0.02455 |
| RP13-582O9.7 | 0.64 (0.43–0.94) | 0.02455 |
| RP11-57H14.4 | 1.65 (1.07–2.54) | 0.02455 |
| RP11-463O12.5 | 1.47 (1.05–2.06) | 0.02459 |
| RP11-444D3.1 | 1.83 (1.08–3.11) | 0.02462 |
| OSBPL10-AS1 | 1.61 (1.06–2.44) | 0.02465 |
| CTC-429P9.2 | 0.61 (0.40–0.94) | 0.02465 |
| RP11-508M8.1 | 1.53 (1.06–2.22) | 0.02474 |
| RP11-78J21.7 | 0.57 (0.34–0.93) | 0.02492 |
| RP3-467L1.6 | 0.67 (0.47–0.95) | 0.02495 |
| RP11-1008C21.2 | 0.64 (0.44–0.95) | 0.02499 |
| CH17-264L24.1 | 1.48 (1.05–2.08) | 0.02515 |
| CTC-459M5.2 | 1.52 (1.05–2.20) | 0.02524 |
| TNKS2-AS1 | 0.65 (0.44–0.95) | 0.02526 |
| RP11-552F3.4 | 1.48 (1.05–2.08) | 0.02537 |
| RP11-620J15.2 | 0.53 (0.30–0.92) | 0.02538 |
| RP11-802O23.3 | 0.68 (0.48–0.95) | 0.02538 |
| SMCR2 | 0.51 (0.28–0.92) | 0.02554 |
| FOXN3-AS1 | 0.44 (0.22–0.90) | 0.02554 |
| RP5-1142A6.2 | 1.73 (1.07–2.79) | 0.02555 |
| RP11-447D11.3 | 1.59 (1.06–2.39) | 0.02555 |
| RP11-381N20.2 | 0.67 (0.47–0.95) | 0.02556 |
| SNHG11 | 1.74 (1.07–2.83) | 0.02566 |
| RP11-771K4.3 | 0.57 (0.35–0.93) | 0.02577 |
| RP11-317N8.3 | 1.68 (1.06–2.66) | 0.02577 |
| AC107057.1 | 0.66 (0.46–0.95) | 0.0259 |
| RP11-849I19.2 | 0.65 (0.45–0.95) | 0.026 |
| AC093642.3 | 1.56 (1.05–2.30) | 0.02617 |
| RP11-348P10.2 | 0.51 (0.28–0.92) | 0.02631 |
| C6orf99 | 0.67 (0.46–0.95) | 0.02651 |
| RP11-118M9.3 | 0.63 (0.41–0.95) | 0.02651 |
| LINC00954 | 0.57 (0.35–0.94) | 0.02666 |
| RP11-97C16.1 | 0.64 (0.43–0.95) | 0.02666 |
| MAPKAPK5-AS1 | 1.64 (1.06–2.55) | 0.02676 |
| RP11-498P14.5 | 0.58 (0.36–0.94) | 0.02679 |
| LA16c-390E6.4 | 1.50 (1.05–2.15) | 0.02679 |
| RP11-295P9.12 | 0.54 (0.32–0.93) | 0.02681 |
| NFIA-AS2 | 1.78 (1.07–2.97) | 0.02683 |
| AC005537.2 | 0.66 (0.45–0.95) | 0.02692 |
| RP11-428O18.6 | 0.63 (0.42–0.95) | 0.02696 |
| CTD-2561F5.1 | 1.81 (1.07–3.07) | 0.02703 |
| SNHG15 | 1.61 (1.06–2.47) | 0.02709 |
| RP11-69E11.8 | 1.64 (1.06–2.53) | 0.02711 |
| SNHG7 | 1.48 (1.05–2.10) | 0.02716 |
| AC005740.6 | 1.52 (1.05–2.21) | 0.02722 |
| AC007036.4 | 1.57 (1.05–2.35) | 0.02728 |
| SNHG23 | 0.44 (0.21–0.91) | 0.02738 |
| CTD-2528A14.5 | 1.61 (1.05–2.45) | 0.02745 |
| TAF1A-AS1 | 0.68 (0.48–0.96) | 0.0276 |
| RP13-131K19.1 | 0.68 (0.49–0.96) | 0.02767 |
| RP11-168O16.1 | 0.67 (0.47–0.96) | 0.02767 |
| CTC-523E23.4 | 2.00 (1.08–3.72) | 0.02781 |
| RNASEH2B-AS1 | 1.58 (1.05–2.38) | 0.02787 |
| RP11-223A3.1 | 0.68 (0.48–0.96) | 0.02811 |
| RP11-661A12.8 | 0.69 (0.49–0.96) | 0.02826 |
| PSMA3-AS1 | 0.61 (0.39–0.95) | 0.02834 |
| AC068831.16 | 0.68 (0.48–0.96) | 0.02841 |
| AP000648.5 | 0.62 (0.40–0.95) | 0.02842 |
| SHANK3 | 1.50 (1.04–2.15) | 0.02847 |
| LINC01023 | 0.61 (0.40–0.95) | 0.02854 |
| RP11-755B10.4 | 0.67 (0.47–0.96) | 0.02865 |
| RP11-244M2.1 | 0.66 (0.46–0.96) | 0.02875 |
| TPT1-AS1 | 1.50 (1.04–2.14) | 0.02879 |
| RP11-584P21.2 | 0.68 (0.49–0.96) | 0.02885 |
| RP5-1112D6.8 | 1.46 (1.04–2.04) | 0.02886 |
| RP11-396C23.4 | 0.68 (0.48–0.96) | 0.02887 |
| RP11-168F9.2 | 1.70 (1.06–2.74) | 0.02895 |
| RP11-326A19.3 | 0.51 (0.28–0.93) | 0.02909 |
| CTB-119C2.1 | 0.63 (0.42–0.95) | 0.02909 |
| CTD-2270L9.4 | 0.59 (0.37–0.95) | 0.02924 |
| RP11-231G3.1 | 1.72 (1.06–2.80) | 0.02924 |
| RP11-264B17.2 | 0.59 (0.37–0.95) | 0.0293 |
| RP11-506M12.1 | 0.66 (0.45–0.96) | 0.02944 |
| CTD-2249K22.1 | 0.57 (0.35–0.95) | 0.02956 |
| RP11-455O6.9 | 1.47 (1.04–2.07) | 0.02958 |
| RP11-1376P16.2 | 1.69 (1.05–2.70) | 0.02964 |
| LLNLR-304G9.1 | 0.60 (0.38–0.95) | 0.02968 |
| RP11-35G9.3 | 0.69 (0.49–0.96) | 0.02976 |
| CTC-265F19.1 | 1.48 (1.04–2.12) | 0.02987 |
| RP5-1184F4.5 | 1.76 (1.06–2.94) | 0.02992 |
| KB-318B8.7 | 1.46 (1.04–2.06) | 0.02997 |
| CTD-2340E1.2 | 1.45 (1.04–2.04) | 0.03009 |
| RP3-522D1.1 | 0.64 (0.43–0.96) | 0.03012 |
| RP11-68I18.10 | 1.45 (1.04–2.04) | 0.03018 |
| RP11-132F7.2 | 0.63 (0.41–0.96) | 0.03023 |
| RP11-170M17.1 | 1.67 (1.05–2.64) | 0.03027 |
| TSC22D1-AS1 | 1.62 (1.05–2.50) | 0.0303 |
| AC011997.1 | 0.61 (0.39–0.95) | 0.03047 |
| RP11-334C17.5 | 1.79 (1.06–3.02) | 0.03079 |
| AC092669.6 | 0.62 (0.40–0.96) | 0.03081 |
| RP11-562A8.5 | 1.66 (1.05–2.62) | 0.03086 |
| CTC-786C10.1 | 1.47 (1.04–2.09) | 0.0312 |
| RP11-384K6.6 | 1.57 (1.04–2.36) | 0.03133 |
| PCAT6 | 0.69 (0.49–0.97) | 0.0316 |
| RP11-75C10.7 | 0.59 (0.37–0.96) | 0.03164 |
| RP11-2B6.2 | 1.45 (1.03–2.04) | 0.03183 |
| RP11-290L1.5 | 1.48 (1.03–2.11) | 0.0319 |
| RP3-325F22.5 | 0.69 (0.49–0.97) | 0.03196 |
| AC007038.7 | 1.69 (1.05–2.72) | 0.03224 |
| AC145343.2 | 0.59 (0.36–0.96) | 0.03231 |
| RP11-357N13.3 | 1.53 (1.04–2.25) | 0.03247 |
| RP11-404E16.1 | 1.97 (1.06–3.66) | 0.03251 |
| CTC-550B14.7 | 1.52 (1.04–2.23) | 0.03263 |
| AC002064.7 | 0.53 (0.30–0.95) | 0.03267 |
| CTC-510F12.7 | 0.52 (0.29–0.95) | 0.03269 |
| RABGAP1L-IT1 | 0.68 (0.48–0.97) | 0.03302 |
| RP4-781B1.5 | 2.44 (1.07–5.55) | 0.03312 |
| RP11-164P12.4 | 1.45 (1.03–2.05) | 0.03321 |
| AC007292.3 | 1.63 (1.04–2.57) | 0.03324 |
| CTD-2517O10.6 | 0.69 (0.49–0.97) | 0.0334 |
| RP11-855A2.3 | 0.56 (0.32–0.95) | 0.03341 |
| AC005330.2 | 1.69 (1.04–2.75) | 0.03362 |
| RP11-347I19.7 | 1.48 (1.03–2.13) | 0.03363 |
| TUG1 | 0.66 (0.45–0.97) | 0.0337 |
| RP1-166H1.2 | 0.48 (0.24–0.94) | 0.03372 |
| RP11-169E6.1 | 0.68 (0.48–0.97) | 0.03382 |
| RP11-257O5.2 | 0.63 (0.41–0.97) | 0.03384 |
| RP11-64K12.8 | 1.83 (1.05–3.19) | 0.03409 |
| ZNF638-IT1 | 1.50 (1.03–2.17) | 0.03412 |
| RP11-329B9.5 | 0.58 (0.35–0.96) | 0.03415 |
| RP11-43N16.4 | 1.47 (1.03–2.09) | 0.03421 |
| AC010524.4 | 1.52 (1.03–2.25) | 0.03421 |
| RP3-393E18.2 | 1.84 (1.05–3.24) | 0.03423 |
| RP11-214K3.20 | 0.56 (0.33–0.96) | 0.0344 |
| RP11-59H7.3 | 0.69 (0.49–0.97) | 0.03457 |
| CTD-2013N17.6 | 1.73 (1.04–2.89) | 0.03463 |
| CTD-2017D11.2 | 0.66 (0.45–0.97) | 0.03464 |
| RP11-385F5.5 | 0.69 (0.49–0.97) | 0.0348 |
| AC004854.4 | 0.68 (0.48–0.97) | 0.03481 |
| AF127577.8 | 0.50 (0.26–0.95) | 0.03501 |
| ZSWIM8-AS1 | 1.55 (1.03–2.32) | 0.03509 |
| UBE2E1-AS1 | 0.69 (0.49–0.97) | 0.03512 |
| AC092301.3 | 1.51 (1.03–2.22) | 0.03512 |
| CTC-546K23.1 | 0.53 (0.29–0.96) | 0.0352 |
| RP11-102N12.3 | 0.59 (0.36–0.96) | 0.03527 |
| AC062017.1 | 0.67 (0.46–0.97) | 0.03549 |
| RP11-342K6.2 | 1.44 (1.02–2.01) | 0.0355 |
| RP11-27N21.3 | 0.67 (0.47–0.97) | 0.0356 |
| RP1-101A2.1 | 0.67 (0.47–0.97) | 0.03561 |
| RP11-217B1.2 | 0.58 (0.35–0.96) | 0.03572 |
| RP11-152F13.8 | 0.67 (0.46–0.97) | 0.03576 |
| RP11-154B12.3 | 0.58 (0.34–0.96) | 0.03578 |
| PRKAG2-AS1 | 1.59 (1.03–2.46) | 0.03579 |
| GSN-AS1 | 1.56 (1.03–2.35) | 0.0359 |
| RP11-286H14.8 | 1.51 (1.03–2.22) | 0.03596 |
| AC107081.5 | 1.44 (1.02–2.03) | 0.03602 |
| LINC01257 | 0.64 (0.42–0.97) | 0.03615 |
| RP11-298J20.3 | 0.53 (0.29–0.96) | 0.03616 |
| RP11-462B18.2 | 1.57 (1.03–2.41) | 0.03623 |
| HYMAI | 1.44 (1.02–2.03) | 0.03632 |
| SNCA-AS1 | 1.45 (1.02–2.06) | 0.03649 |
| KB-1125A3.11 | 0.68 (0.47–0.98) | 0.03652 |
| AC007193.10 | 1.78 (1.04–3.04) | 0.03654 |
| CTD-3157E16.2 | 0.67 (0.46–0.98) | 0.03656 |
| LINC01359 | 1.62 (1.03–2.54) | 0.03662 |
| RP11-74J13.8 | 1.54 (1.03–2.30) | 0.03668 |
| CTC-458I2.2 | 0.53 (0.29–0.96) | 0.03678 |
| RP11-620J15.4 | 0.69 (0.48–0.98) | 0.03683 |
| RP11-169K16.4 | 0.63 (0.41–0.97) | 0.03697 |
| RP11-54O7.2 | 0.70 (0.50–0.98) | 0.03702 |
| RP11-179B2.2 | 0.56 (0.33–0.97) | 0.03705 |
| RP5-875H18.10 | 0.67 (0.46–0.98) | 0.03707 |
| RP11-549B18.1 | 0.63 (0.40–0.97) | 0.03707 |
| FAM225B | 1.80 (1.04–3.14) | 0.03715 |
| LINC00853 | 0.64 (0.42–0.97) | 0.03719 |
| RP11-61L19.1 | 0.57 (0.34–0.97) | 0.03724 |
| RP11-573D15.9 | 0.67 (0.47–0.98) | 0.03727 |
| RP11-84C10.4 | 0.62 (0.39–0.97) | 0.0373 |
| RP11-145M9.6 | 1.49 (1.02–2.18) | 0.03752 |
| RP11-363D14.1 | 0.53 (0.29–0.96) | 0.03763 |
| GS1-124K5.13 | 1.66 (1.03–2.67) | 0.03772 |
| RP11-767N6.7 | 0.52 (0.28–0.96) | 0.0379 |
| RP11-527J8.1 | 0.63 (0.41–0.97) | 0.03803 |
| RP11-783K16.5 | 0.69 (0.48–0.98) | 0.03811 |
| AC135048.13 | 0.69 (0.48–0.98) | 0.03826 |
| RP5-892K4.1 | 1.50 (1.02–2.20) | 0.03829 |
| C9orf147 | 0.66 (0.44–0.98) | 0.03829 |
| RP11-517H2.6 | 1.51 (1.02–2.23) | 0.03841 |
| RP5-1125A11.7 | 0.70 (0.50–0.98) | 0.03845 |
| RP13-516M14.1 | 0.58 (0.35–0.97) | 0.03846 |
| RP11-513O13.1 | 0.68 (0.48–0.98) | 0.03859 |
| CTB-113P19.5 | 1.72 (1.03–2.88) | 0.03863 |
| LINC00680 | 0.66 (0.45–0.98) | 0.0388 |
| RP11-517B11.7 | 1.92 (1.03–3.57) | 0.03884 |
| CTC-523E23.11 | 0.70 (0.49–0.98) | 0.03887 |
| RGMB-AS1 | 0.70 (0.50–0.98) | 0.03907 |
| CTC-563A5.2 | 1.72 (1.03–2.89) | 0.03914 |
| RP11-3D4.3 | 0.61 (0.38–0.98) | 0.03916 |
| CTD-2591A6.2 | 0.66 (0.45–0.98) | 0.03918 |
| RP11-13K12.1 | 1.44 (1.02–2.05) | 0.03918 |
| RP11-386G11.10 | 0.63 (0.40–0.98) | 0.03931 |
| RP11-299J3.8 | 0.68 (0.47–0.98) | 0.03935 |
| CTD-2015H6.3 | 0.64 (0.41–0.98) | 0.03942 |
| C20orf166-AS1 | 1.74 (1.03–2.93) | 0.0395 |
| AC096772.6 | 1.44 (1.02–2.04) | 0.03966 |
| RP11-506H21.5 | 0.68 (0.47–0.98) | 0.03971 |
| RP4-533D7.5 | 0.64 (0.42–0.98) | 0.03986 |
| AC114271.2 | 0.68 (0.47–0.98) | 0.03995 |
| RP11-200A1.1 | 0.55 (0.31–0.97) | 0.04005 |
| CTC-367J11.1 | 0.67 (0.46–0.98) | 0.04007 |
| RP11-403A3.3 | 0.69 (0.49–0.98) | 0.04016 |
| ENO1-AS1 | 0.61 (0.38–0.98) | 0.0402 |
| AL132709.8 | 0.68 (0.47–0.98) | 0.04055 |
| LINC01431 | 0.69 (0.48–0.98) | 0.0409 |
| RP11-977G19.5 | 1.63 (1.02–2.60) | 0.04093 |
| SPG20-AS1 | 0.70 (0.50–0.99) | 0.0412 |
| RP11-736K20.6 | 0.55 (0.31–0.98) | 0.04131 |
| RP11-818O24.2 | 1.43 (1.01–2.00) | 0.04134 |
| CTD-2024I7.18 | 0.67 (0.46–0.98) | 0.04135 |
| RP11-533E19.7 | 0.70 (0.49–0.99) | 0.04138 |
| CTD-2270L9.3 | 1.44 (1.01–2.04) | 0.04161 |
| RP11-478K15.6 | 1.42 (1.01–2.00) | 0.04167 |
| ZNF32-AS2 | 0.69 (0.48–0.99) | 0.04168 |
| RP11-554J4.1 | 0.70 (0.50–0.99) | 0.04173 |
| CTD-2555K7.2 | 0.67 (0.46–0.99) | 0.04195 |
| RP11-180P8.1 | 1.45 (1.01–2.09) | 0.04202 |
| RP1-59D14.5 | 1.61 (1.02–2.54) | 0.04207 |
| YEATS2-AS1 | 1.57 (1.02–2.44) | 0.04228 |
| RP11-293A21.1 | 0.70 (0.50–0.99) | 0.04228 |
| RP11-379K22.2 | 0.55 (0.31–0.98) | 0.04232 |
| ELOVL2-AS1 | 0.53 (0.28–0.98) | 0.0424 |
| RP11-86K22.2 | 1.50 (1.01–2.21) | 0.04254 |
| CTC-524C5.2 | 1.43 (1.01–2.02) | 0.04255 |
| IQCH-AS1 | 0.69 (0.49–0.99) | 0.04259 |
| CTD-2588C8.8 | 0.70 (0.49–0.99) | 0.04271 |
| RP11-375O18.2 | 0.55 (0.31–0.98) | 0.04331 |
| RP11-383M4.6 | 0.63 (0.41–0.99) | 0.0435 |
| HCG15 | 0.69 (0.48–0.99) | 0.04358 |
| CITF22-92A6.1 | 1.72 (1.02–2.90) | 0.0438 |
| KB-1836B5.4 | 0.70 (0.50–0.99) | 0.04386 |
| RP1-167A14.2 | 1.43 (1.01–2.02) | 0.04389 |
| RP11-342A23.2 | 0.56 (0.32–0.98) | 0.04404 |
| TRAF3IP2-AS1 | 1.42 (1.01–2.01) | 0.04413 |
| RP13-16H11.2 | 1.51 (1.01–2.26) | 0.04421 |
| RP11-486L19.2 | 0.69 (0.47–0.99) | 0.0444 |
| RP11-335O4.3 | 1.42 (1.01–2.01) | 0.04455 |
| L29074.3 | 0.70 (0.49–0.99) | 0.04481 |
| AC019048.1 | 0.57 (0.32–0.99) | 0.04497 |
| RP11-191L17.1 | 0.66 (0.44–0.99) | 0.04499 |
| RP11-667K14.14 | 1.51 (1.01–2.27) | 0.04509 |
| RP11-33O4.1 | 1.83 (1.01–3.32) | 0.04515 |
| RP11-379F4.6 | 1.56 (1.01–2.41) | 0.04516 |
| CACNA1C-AS2 | 1.41 (1.01–1.98) | 0.04527 |
| CDIPT-AS1 | 0.68 (0.46–0.99) | 0.04535 |
| RP11-87H9.4 | 1.45 (1.01–2.08) | 0.04548 |
| C17orf100 | 0.57 (0.32–0.99) | 0.04556 |
| PKI55 | 0.58 (0.34–0.99) | 0.04557 |
| AC074117.10 | 0.67 (0.46–0.99) | 0.04568 |
| AC073046.25 | 1.67 (1.01–2.75) | 0.04575 |
| RAB30-AS1 | 0.68 (0.46–0.99) | 0.04578 |
| AC005740.5 | 0.69 (0.48–0.99) | 0.04593 |
| RP11-359K18.4 | 0.67 (0.46–0.99) | 0.04594 |
| CYP4F35P | 1.61 (1.01–2.57) | 0.04594 |
| HOXA-AS3 | 1.42 (1.01–1.99) | 0.04601 |
| AC159540.1 | 1.61 (1.01–2.57) | 0.04602 |
| CTC-303L1.2 | 1.56 (1.01–2.43) | 0.04612 |
| RP11-73M18.7 | 1.80 (1.01–3.20) | 0.04619 |
| KB-431C1.4 | 1.41 (1.01–1.99) | 0.04631 |
| RP11-416H1.2 | 1.41 (1.01–1.98) | 0.04637 |
| AC003075.4 | 0.54 (0.30–0.99) | 0.04658 |
| RP13-20L14.1 | 1.41 (1.01–1.99) | 0.04659 |
| RP11-539G18.3 | 0.69 (0.48–0.99) | 0.0466 |
| RP11-452K12.7 | 0.66 (0.44–0.99) | 0.04664 |
| RP11-389O22.1 | 1.66 (1.01–2.75) | 0.04669 |
| CTB-176F20.3 | 1.60 (1.01–2.56) | 0.04673 |
| RP4-734G22.3 | 1.42 (1.01–2.02) | 0.04679 |
| RP11-357N13.2 | 1.42 (1.00–2.00) | 0.0469 |
| CTA-204B4.2 | 0.65 (0.43–0.99) | 0.04709 |
| RP11-178L8.9 | 1.41 (1.00–1.98) | 0.04709 |
| ADD3-AS1 | 0.59 (0.35–0.99) | 0.04729 |
| RP11-671J11.5 | 1.43 (1.00–2.05) | 0.04735 |
| RP1-224A6.3 | 0.69 (0.47–1.00) | 0.04736 |
| RP11-400L8.2 | 0.68 (0.47–1.00) | 0.04752 |
| RP11-798G7.7 | 1.41 (1.00–1.98) | 0.04754 |
| RP11-395E19.2 | 1.62 (1.01–2.61) | 0.04757 |
| CTD-2576F9.2 | 1.66 (1.01–2.73) | 0.04775 |
| RP11-855A2.1 | 0.65 (0.42–1.00) | 0.04776 |
| SNHG16 | 0.71 (0.51–1.00) | 0.04779 |
| RP11-411B10.7 | 0.60 (0.37–1.00) | 0.04786 |
| RP11-797A18.6 | 0.63 (0.40–1.00) | 0.04792 |
| RP13-554M15.7 | 1.57 (1.00–2.47) | 0.04806 |
| RP1-117O3.2 | 1.41 (1.00–1.98) | 0.04826 |
| RP11-803D5.1 | 0.60 (0.37–1.00) | 0.04838 |
| RP11-1379J22.5 | 0.55 (0.30–1.00) | 0.04914 |
| CTBP1-AS | 1.40 (1.00–1.97) | 0.04925 |
| AC092614.2 | 0.56 (0.32–1.00) | 0.04935 |
| LL22NC03-2H8.4 | 1.43 (1.00–2.03) | 0.04935 |
| CTD-3094K11.1 | 0.69 (0.48–1.00) | 0.04935 |
| KANSL1-AS1 | 0.62 (0.39–1.00) | 0.04953 |
| RP11-510J16.5 | 0.70 (0.50–1.00) | 0.04955 |
| RP11-290H9.5 | 0.64 (0.41–1.00) | 0.04957 |
| XXbac-BPG283O16.9 | 1.57 (1.00–2.46) | 0.04959 |
| RP11-77K12.9 | 0.71 (0.50–1.00) | 0.0497 |
| MIR181A2HG | 0.70 (0.50–1.00) | 0.04983 |
| RP11-277A4.4 | 1.60 (1.00–2.55) | 0.04991 |

**Supplementary Table 5. Results of prediction of lncRNA-drug interactions.**

| Drugs | LncRNA | Correlation coefficient |  | P-value |
| --- | --- | --- | --- | --- |
| 17-AAG | RP11-81H14.2 | 0.222 |  | 0.0000653 |
| PD-0325901 | RP11-81H14.2 | 0.212 |  | 0.000143 |
| AZD0530 | RP11-81H14.2 | 0.211 |  | 0.000151 |
| PD-0332991 | RP11-81H14.2 | -0.206 |  | 0.000603 |
| Panobinostat | RP11-81H14.2 | -0.185 |  | 0.000977 |
| ZD-6474 | RP11-81H14.2 | 0.183 |  | 0.00116 |
| Lapatinib | RP11-81H14.2 | 0.174 |  | 0.00185 |
| AZD6244 | RP11-81H14.2 | 0.171 |  | 0.00224 |
| AZD6244 | AC072062.3 | 0.154 |  | 0.00614 |
| PD-0325901 | AC072062.3 | 0.152 |  | 0.00674 |
| Topotecan | RP11-81H14.2 | -0.144 |  | 0.0103 |
| LBW242 | RP11-81H14.2 | 0.142 |  | 0.0114 |
| Panobinostat | MYCNOS | -0.142 |  | 0.0116 |
| PF2341066 | RP11-81H14.2 | -0.14 |  | 0.0126 |
| AZD0530 | RP11-1109M24.5 | 0.137 |  | 0.0144 |
| Lapatinib | RP13-476E20.1 | -0.136 |  | 0.0154 |
| Erlotinib | RP11-81H14.2 | 0.133 |  | 0.018 |
| PLX4720 | RP13-476E20.1 | 0.133 |  | 0.019 |
| L-685458 | RP11-81H14.2 | -0.125 |  | 0.0286 |
| PD-0325901 | RP11-508M8.1 | 0.12 |  | 0.0327 |
| AZD6244 | RP11-508M8.1 | 0.118 |  | 0.0353 |
| Nutlin-3 | RP11-81H14.2 | -0.118 |  | 0.0353 |
| Irinotecan | RP11-81H14.2 | -0.142 |  | 0.0414 |
| Lapatinib | RP11-1109M24.5 | 0.114 |  | 0.042 |
| Lapatinib | RP11-508M8.1 | 0.114 |  | 0.0423 |
| Erlotinib | RP13-476E20.1 | -0.114 |  | 0.0428 |


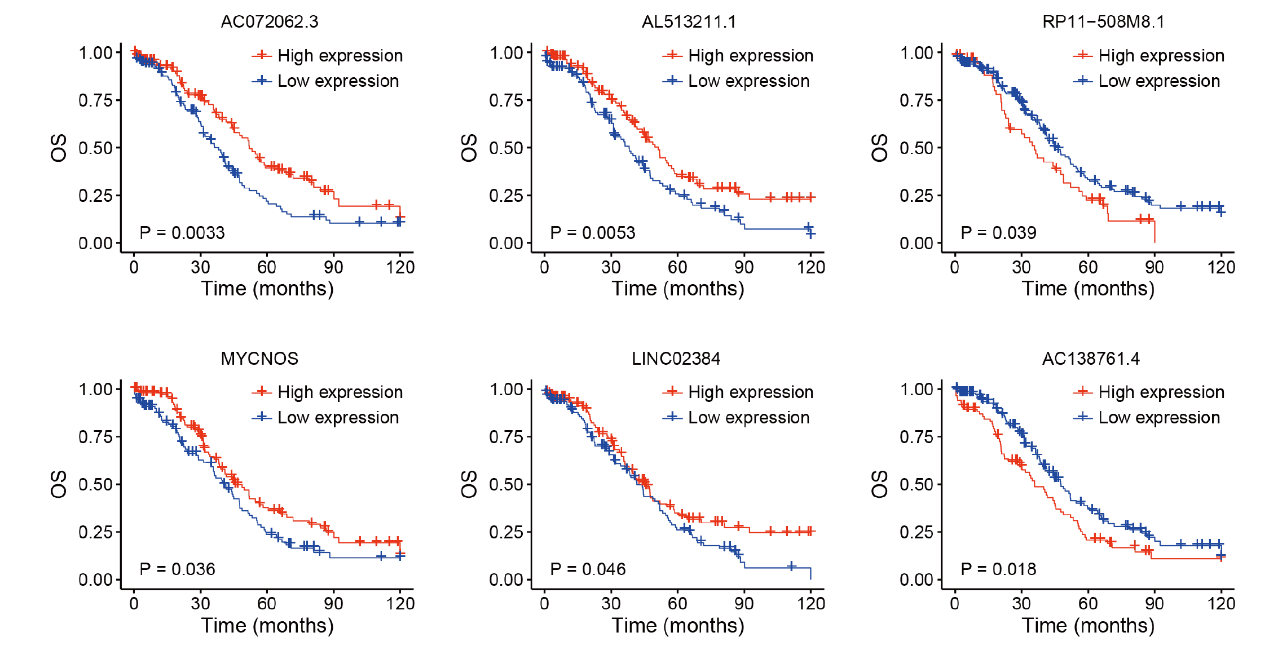


**Supplementary Figure 1. KM survival analysis of m^6^A effector-related lncRNAs in the training set.** Kaplan–Meier (KM); overall survival (OS)

**
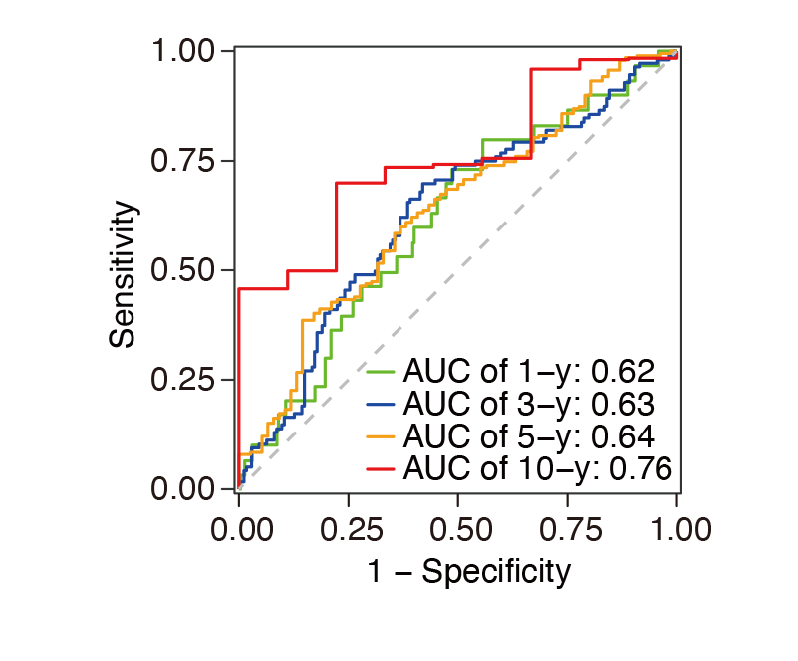
**

**Supplementary Figure 2. Assessment of prognostic value of the risk model.** ROC curves of m^6^A-LRM within 1-, 3-, 5-, and 10-year survival periods, revealing the stable evaluation ability. m^6^A effector-related lncRNA risk model (m^6^A-LRM); Receiver operating characteristic (ROC)

**
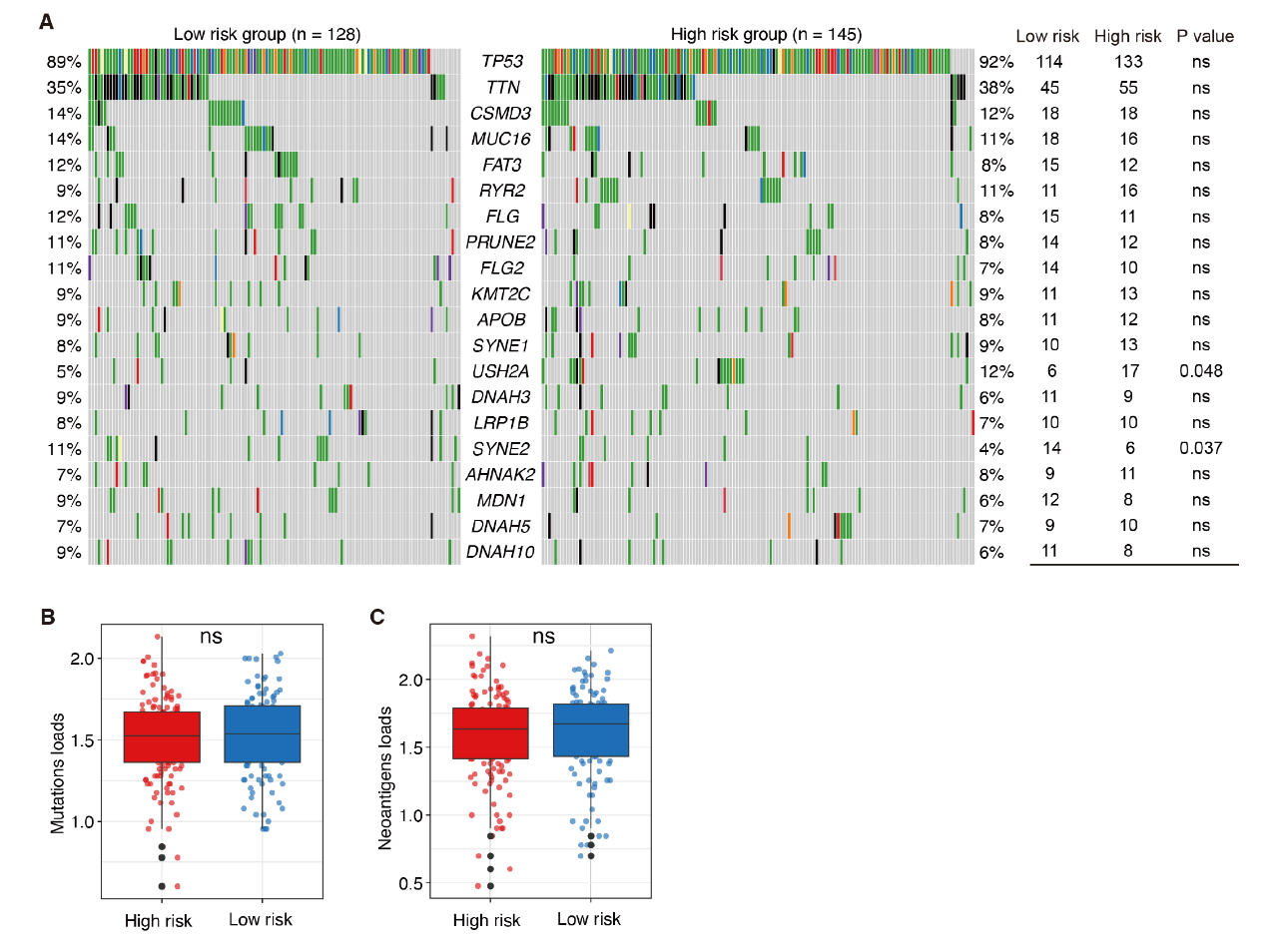
**

**Supplementary Figure 3. Mutational landscape for the m^6^A-LRM in SOC. (A)** Waterfall plot displaying the top 20 genes with high mutational frequencies. **(B-C)** Mutation and neoantigen loads between the two subgroups are displayed. m^6^A effector-related lncRNA risk model (m^6^A-LRM); serous ovarian carcinoma (SOC)

**
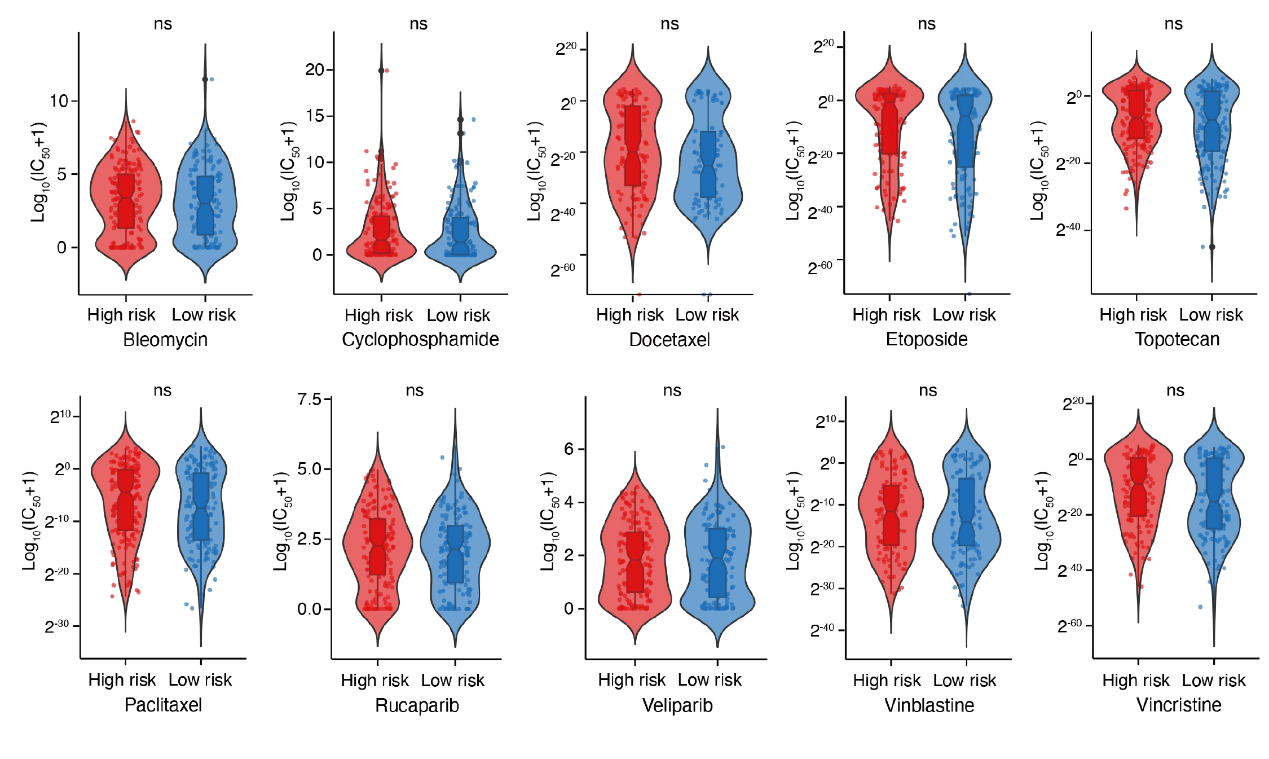
**

**Supplementary** **Figure 4. Drug sensitivity evaluation of ten common drugs for serous ovarian carcinoma (SOC).** The Y-axis represents the half maximal inhibitory concentration (IC_50_). ns, not significant.
